# Laser therapy decreases oral leukoplakia recurrence and boosts patient comfort: a network meta-analysis and systematic review

**DOI:** 10.1186/s12903-024-04179-9

**Published:** 2024-04-17

**Authors:** Rui Luo, Yanan Wang, Ruixin Li, Yanan Ma, Haitao Chen, Jian Zhang, Jun Shen

**Affiliations:** 1 National Clinical Research Center for Cancer, Tianjin’s Clinical Research Center for Cancer, Tianjin Medical University Cancer Institute & Hospital, Tianjin, 300060 China; 2 Tianjin Medical University, Tianjin, 300070 China; 3 School of Stomatology, Hospital of Stomatology, Tianjin Medical University, 12 Observatory Road, Tianjin, 300070 China; 4 Tianjin Key Laboratory of Oral and Maxillofacial Function Reconstruction, Tianjin Stomatological Hospital, Hospital of Stomatological, Nankai University, 75 Dagu Road, Heping District, Tianjin, 300041 China; 5 School of Stomatology, Weifang Medical University, Weifang, 261053 China

**Keywords:** Oral leukoplakia, Precancerous lesion, Laser, Photodynamic therapy, Network meta-analysis

## Abstract

**Background:**

Oral leukoplakia (OLK) is a prevalent precancerous lesion with limited non-pharmacological treatment options. Surgery and various lasers are the mainstay of treatment; however, their relative efficacy and optimal choice remain unclear. This first network meta-analysis compared the effects of different lasers and surgical excision on post-treatment recurrence and comfort in OLK patients.

**Methods:**

We searched four databases for relevant randomized controlled trials (RCTs) up to April 2023. The primary outcome was post-treatment recurrence, and secondary outcomes included intraoperative hemorrhage and postoperative pain scores. The Cochrane Risk of Bias tool was used to assess the study quality. Meta-analysis and network meta-analysis were employed to determine efficacy and identify the optimal intervention.

**Results:**

A total of 11 RCTs including 917 patients and 1138 lesions were included. Er,Cr:YSGG laser treatment showed significantly lower recurrence rates compared to CO_2_ laser (OR: 0.04; 95% CI: 0.01–0.18), CO_2_ laser with margin extension (OR: 0.06; 95% CI: 0.01–0.60), Er:YAG laser (OR: 0.10; 95% CI: 0.03–0.37), electrocautery (OR: 0.03; 95% CI: 0.00–0.18), and standard care (OR: 0.08; 95% CI: 0.02–0.33). Er,Cr:YSGG laser also ranked the best for reducing recurrence, followed by standard care and CO_2_ laser combined with photodynamic therapy (PDT). Er:YAG and Er:Cr:YSGG lasers minimized bleeding and pain, respectively. None of the interventions caused severe adverse effects.

**Conclusion:**

For non-homogeneous OLK, Er:YAG, Er:Cr:YSGG, and CO_2_ laser combined with PDT offer promising alternatives to surgical excision, potentially reducing recurrence and improving patient comfort. Further high-quality RCTs are necessary to confirm these findings and determine the optimal laser–PDT combination for OLK treatment.

**Supplementary Information:**

The online version contains supplementary material available at 10.1186/s12903-024-04179-9.

## Introduction

Oral leukoplakia (OLK) is a common oral mucosal disease characterized by predominantly white lesions on the oral mucosa [1]. These lesions cannot be definitively diagnosed like other specific diseases through clinical and histopathological methods. OLK is a precancerous lesion that can potentially evolve into oral squamous cell carcinoma (OSCC) [2]. Recurrence of OLK after treatment is possible, and these recurrent episodes increase the risk of cancerous lesions, imposing a substantial financial burden on both patients and society [3]. Therefore, appropriate approaches to alleviate OLK symptoms and minimize post-treatment recurrence are crucial for enhancing patients’ quality of life and preventing malignant transformation.

Current treatment options for OLK include medication, surgery, and physical therapy [4]. Although medications such as vitamin A and retinoids have demonstrated treatment efficacy, their lengthy therapeutic use and high individual selection bias might lead to severe liver and kidney toxicity, hyperlipidemia, and fetal malformations [5]. In contrast to drug therapy, surgery is considered the “gold standard” for managing OLK, considering its clear indications, broad applicability, and excellent clinical efficacy [6]. However, surgery involves intraoperative hemorrhage and trauma, with tissue defects and dysfunction, significantly affecting patients’ willingness to visit the clinic and their quality of life. Recent advancements in laser and equipment miniaturization technologies have made lasers a highly targeted, rapid, and minimally invasive therapeutic approach for OLK [7]. Tissue defects and dysfunction induced by lasers are minimal, with little impact on the patient’s postoperative quality of life. Researchers have conducted clinical trials on various lasers, including CO_2_ and ruby lasers, for OLK treatment [8, 9]. However, the magnitude of the advantages of laser treatment over surgical excision remains unclear. Additionally, variations in laser generation and emission principles result in differing effects on the ablation of OLK [10, 11]. Therefore, comparing OLK patients’ different benefits after laser and surgical treatments, analyzing possible differences in the effectiveness of different laser types for OLK, and exploring laser treatments with the most favorable outcomes can provide a sound theoretical basis for informed clinical treatment selection and optimization strategies.

Traditional meta-analysis integrates effect sizes from trials of the same intervention, providing strong and credible evidence for the informed selection of appropriate interventions for a specific disease [12]. Previous meta-analyses have primarily focused on the effect of laser and surgical excision on the malignant process of OLK [13]. However, the factors affecting OLK carcinogenesis are complex, including pathological staging, duration of disease, and the patient’s reasons. It is not accurate to evaluate the efficacy of different interventions for OLK by assessing the malignant transformation rate if the original stimuli are still present and the patient’s lifestyle habits have not changed. Additionally, methodological limitations and the scarcity of randomized controlled trials (RCTs) have hampered attempts to compare different lasers’ specific therapeutic effects on OLK and determine the effects of interventions [14]. Consequently, researchers are increasingly embracing the more comprehensive and integrated approach of network meta-analysis, which innovatively establishes a network for comparing multiple interventions and integrating both direct and indirect evidence to comprehensively evaluate the impact of different interventions [15]. This network structure allows the inclusion of interventions lacking direct comparisons, significantly expanding the available evidence base [16]. Network meta-analysis enables the robust assessment of the relative effectiveness of multiple interventions through multivariate, direct, and indirect comparisons, even when head-to-head trials are absent. This approach provides a more holistic perspective of the available evidence, facilitating the ranking of interventions based on their relative efficacy and often allowing visual representation in network plots [17].

This systematic review and network meta-analysis represents the most recent comparison of various laser and surgical excision techniques for managing OLK. The study’s primary objective was to assess the incidence of recurrence after different interventions for OLK, while the secondary objective was to evaluate the impact of these interventions on patients’ postoperative trauma, pain, and adverse reactions. This study used an integrated network model that combines direct and indirect evidence to provide a comprehensive summary and analysis of the results of various laser and surgical resection treatments for OLK, with the ultimate goal of generating reliable evidence for the informed selection of optimal treatment options for OLK in clinical practice.

## Materials and methods

This systematic review and network meta-analysis focused exclusively on RCTs evaluating the efficacy of various laser and surgical interventions for OLK. The study adhered to the rigorous methodological principles outlined in the Cochrane Handbook for Systematic Reviews of Interventions and followed the Preferred Reporting Items for Systematic Reviews and Meta-Analyses (PRISMA) extension statement for network meta-analysis, ensuring reporting completeness [18, 19]. To further enhance transparency, the systematic review was registered in the PROSPERO database (National Institute for Health and Care Research, CRD42023435477).

### Search strategy

We conducted a comprehensive and systematic search in four major databases: PubMed, EMBASE, Web of Science and the Cochrane Library. The search encompassed a vast timeframe (the time spans of the searches were detailed in Table 1), encompassing articles published in all languages. We restricted our search to published articles with an RCT design to ensure the inclusion of only the highest-quality evidence. The primary search terms were “oral leukoplakia” and “oral leukokeratosis”. In a dedicated effort to capture the most recent findings, we examined ongoing RCTs presented at major conferences organized by the World Dental Federation and the American Dental Association over the past 5 years. Additionally, we meticulously screened the reference lists of all the included studies to identify any missed literature, as detailed in Supplementary Table S1.

**Table 1:** The time spans of the searches

Databases	Start	End
PubMed	1996	April 2023
EMBASE	1946	April 2023
Web of Science	1997	April 2023
Cochrane Library	1993	April 2023

### Inclusion and exclusion criteria

The inclusion criteria for the literature in the search process included the following: RCT study design; completion of the entire course of treatment for OLK by any laser type or surgical procedure; and a study duration of > 12 months or a postoperative follow-up of not < 3 months. Exclusion criteria during the search encompassed the following: case reports, observational studies (including cross-sectional, case-control, and cohort designs), letters, personal opinions, book chapters, duplicate publications, studies with limited sample sizes, studies with ambiguous baselines, and studies with inappropriate reporting of endpoints. These criteria were applied to refine the selection of literature for this study.

The Participants, Intervention, Comparator, and Outcomes (PICO) criteria followed in this study were as follows:

- P: Patients with a confirmed diagnosis of OLK based on both clinical and/or histopathological criteria.
- I: Treatment with any laser type, including but not limited to CO_2_, Er:YAG, and Er:Cr:YSGG lasers.
- C: Any alternative non-pharmacological intervention for OLK, such as surgical excision or removal of causative irritants.
- O: The primary outcome was the number of recurrences, defined as the number of patients experiencing a recurrence of OLK in the same location following each intervention. Secondary outcomes included intraoperative hemorrhage specific to each intervention and quantification of pain on the first postoperative day using a validated numerical pain scoring system.

### Data extraction

Two researchers (Rui Luo and Yanan Wang) independently extracted the following data from the selected studies: study title and primary author, sample size, gender composition, mode of intervention, lesion characteristics (number, location, and nature), study and follow-up durations, and primary and secondary outcome indicators. The most recent data were incorporated in cases where multiple publications stemmed from the same trial. Any discrepancies arising during data extraction were resolved through a discussion between Rui Luo and Yanan Wang. If necessary, a third researcher, Ruixin Li, was consulted to facilitate consensus and ensure data accuracy.

### Quality assessment

Two independent researchers, Yanan Ma and Haitao Chen, evaluated the quality of the included studies using the Cochrane Risk of Bias tool. This tool assesses the risk of bias across six domains: selection bias, performance bias, detection bias, attrition bias, reporting bias, and other potential sources of bias [20]. Disagreements arising during the assessment process were resolved by a third researcher, Ruixin Li, to ensure consensus and minimize the risk of bias. Risk of bias maps were then generated using Review Manager 5.3 software to visually summarize the assessment findings.

### Data integration and analysis

The three most frequently reported outcome measures in the included studies were the number of postoperative recurrences, intraoperative hemorrhage, and pain scores on the first postoperative day. Consequently, these three measures were used as the primary indicators for evaluating the effectiveness of each therapeutic method. Other potential outcome metrics, such as the duration of the operation and quality of life scores after surgery, were not consistently reported in the analyzed studies. Therefore, they were not included in the data extraction, compilation, and analysis.

Concerning the outcomes reported by at least three studies under the same comparison, a classical meta-analysis was conducted using Stata software version 15.0 (USA). Concerning dichotomous data, such as the number of recurrences, odds ratios (ORs) and their corresponding 95% confidence intervals (95% CI) were calculated as summary statistics for effect sizes. These ORs were calculated from the number of patients with and without recurrence in each group. Concerning continuous data, similar to postoperative pain scores, standardized mean differences (SMDs) and their respective 95% CIs were calculated as summary statistics for effect sizes. The means and standard deviations for these continuous outcomes were either directly extracted from the studies or calculated based on published data calculations. Statistical significance was established at *p*-value < 0.05.

Heterogeneity between studies was assessed using both Q-tests and I^2^ values. Based on the magnitude of the I^2^ value, a fixed-effects model or a random-effects model was selected for meta-analysis [21]. A random-effects model was employed when I^2^ was > 50%, indicating significant heterogeneity. Conversely, a fixed-effects model was used when I^2^ was ≤50%, suggesting minimal heterogeneity [22].

Network meta-analysis was performed using the network package of Stata software version 15.0 (USA), employing a frequency theory-based approach. Network diagrams were drawn for each outcome event to visually represent the network structure and connections between interventions. The inconsistency factor (IF) and its 95% CI were derived using a z-test to assess potential discrepancies between direct and indirect comparisons within the network. An IF value close to zero and a 95% CI containing zero indicate consistency between direct and indirect estimates [23, 24]. Funnel plots were constructed to assess the presence of small study effects or publication bias [25]. To summarize the overall effect sizes and their uncertainties, forest plots were generated for the reticulated meta-analyses, displaying the total effect sizes for all the comparisons alongside their 95% confidence and prediction intervals. Areas under the cumulative ranking curve (SUCRA) were calculated to rank the treatment effects of different interventions. Interventions with higher SUCRA values or smaller-ranking values were deemed to have the best treatment effects.

## Results

### Identification of studies

**Fig. 1:**
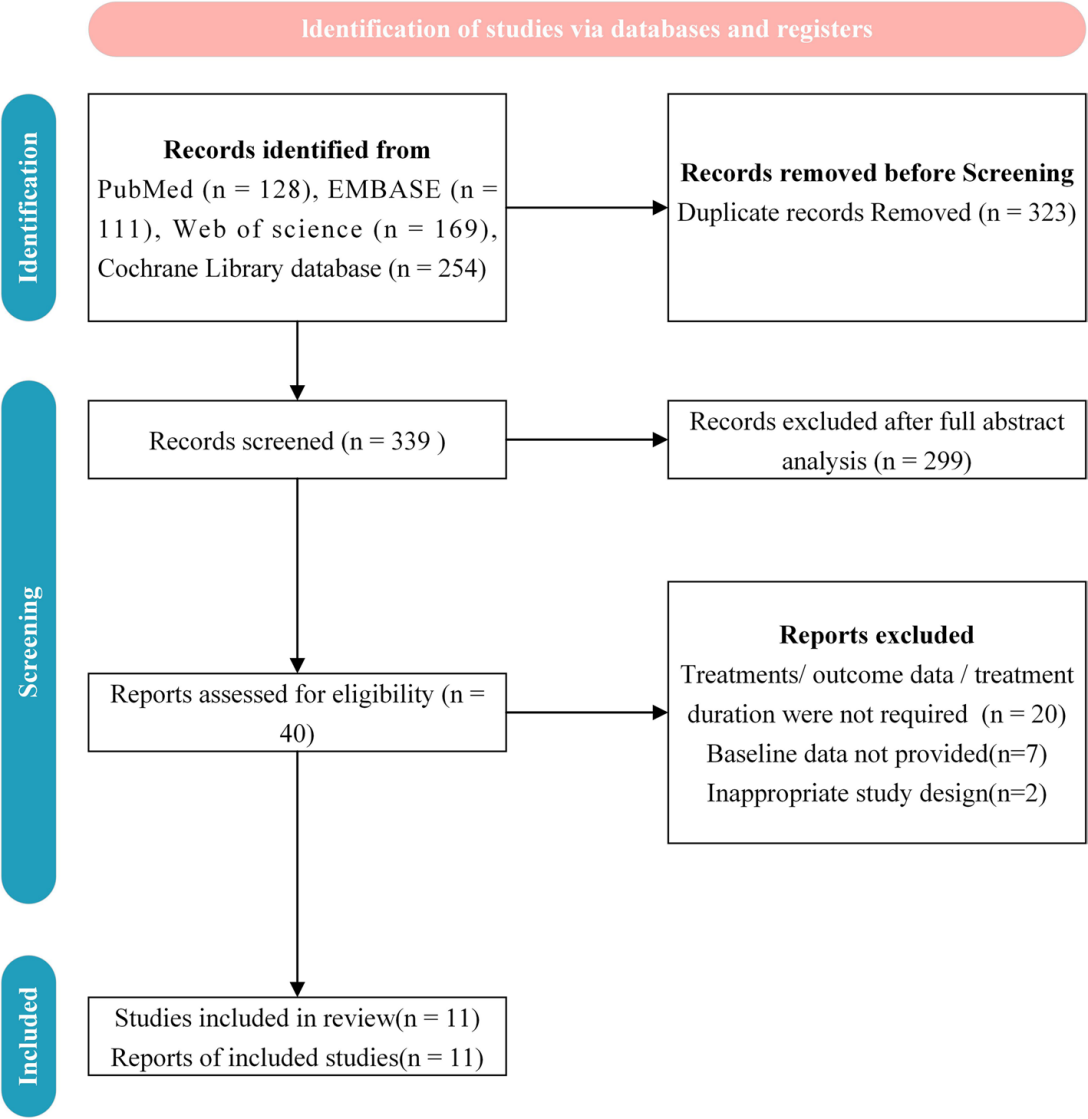
Flow diagram of the included studies

Figure 1 depicts the study retrieval, screening, and final decision-making processes, adhering to the PRISMA guidelines. The initial database search yielded 662 potentially relevant studies. After removing duplicates and reviewing abstracts, 40 studies were deemed eligible for further evaluation. 29 studies were excluded after a thorough examination of the full texts. The reasons for exclusion included treatments, outcome data, or treatment duration not meeting the pre-specified criteria in 20 studies, missing baseline data in 7 studies, and inappropriate study design in 2 studies. Ultimately, 11 RCTs were included in the network meta-analysis. The kappa consistency coefficient (k) between the two researchers, Rui Luo and Yanan Wang, during data extraction was 0.91, indicating good agreement.

### Study characteristics

All the included studies focused on patients with OLK confirmed by clinical and histopathological examinations. Supplementary Table S2 summarizes the general characteristics of the studies. The 11 RCTs encompassed 917 patients with 1138 OLK lesions [26–36]. The longest study duration was 84 months of the reported lesions; 56% (636) were homogeneous OLK, and 14 were non-homogeneous. Three studies did not specify the lesion typology. The most frequent location of OLK was the buccal mucosa, with 339 cases. Eight different interventions were evaluated. Table 2 shows the distribution of treatments.

**Table 2:** The distribution of treatments

Interventions	Number
CO_2_ laser	140
CO_2_ laser extended excision (≥3 mm margin)	11
CO_2_ laser + photodynamic therapy (PDT)	23
Er:YAG laser	292
Er:Cr:YSGG laser	27
Electrocautery	30
Irritant removal (standard care)	130
Conventional surgery	485

### Pairwise meta-analysis

Three pairwise comparisons were identified among the included studies. Supplementary Fig. S1 presents the results. Concerning the number of recurrences in patients with OLK after different treatments, no significant differences were observed between surgical excision and CO_2_ laser therapy (OR, 0.54; 95% CI, 0.27–1.08) or between surgery and Er:YAG laser therapy (OR, 1.26; 95% CI, 0.86–1.83). Regarding intraoperative hemorrhage between treatment modalities, CO_2_ laser demonstrated significantly lower hemorrhage than surgical excision (SMD, 1.11; 95% CI, 0.76–1.47).

### Network meta-analysis

A network meta-analysis was performed to evaluate the number of recurrences, intraoperative hemorrhage, and postoperative pain scores on the first postoperative day after different interventions. The analysis aimed to test for inconsistency at the global level for each outcome separately. None of the *p*-values indicated significant differences, and the test for local inconsistency also showed no significant differences between treatments (Supplementary Tables S3 - S5). The consistency assumption was upheld due to the absence of inconsistency in both global and local tests.

**Fig. 2:**
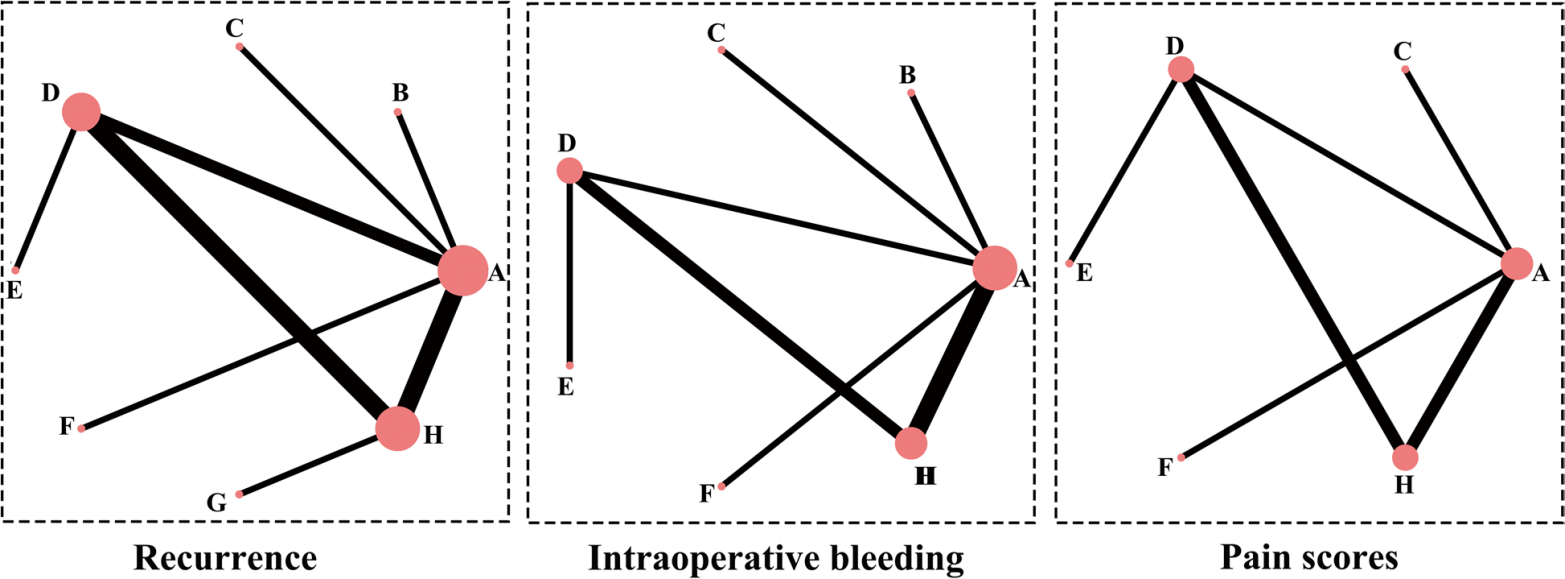
Network map for all outcomes. The size of each node represents the total number of participants receiving that intervention, while the thickness of the lines indicates the number of included studies. (Abbreviations: A: CO_2_ laser, B: CO_2_ laser with margin extension, C: CO_2_ laser + PDT, D: Er: YAG laser, E: Er, Cr:YSGG laser, F: Electrocautery, G: Standard care, H: Surgical excision)

Figure 2 presents network maps for each outcome event. Each node’s size represents the total number of participants undergoing that intervention, while each line’s thickness indicates the number of included studies. Closed loops are formed in each network, with all studies closely interconnected within the loops. Supplementary Fig. S2 displays the results of the loop inconsistency test. All the IF values are close to zero, with their respective 95% confidence intervals including the numeric value 0, indicating no inconsistency between direct and indirect comparisons within the loops.

#### Comparison of the number of recurrences after different interventions

Supplementary Fig. S3 and Table S6 present the results of the primary outcome (number of recurrences) and pairwise comparisons. Statistically significant differences were observed in 11 out of 28 pairwise comparisons. Notably, Er,Cr:YSGG laser treatment demonstrated significantly lower recurrence rates compared to CO_2_ laser (OR: 0.04; 95% CI: 0.01–0.18), CO_2_ laser with margin extension (OR: 0.06; 95% CI: 0.01–0.60), Er:YAG laser (OR: 0.10; 95% CI: 0.03–0.37), electrocautery (OR: 0.03; 95% CI: 0.00–0.18), and standard care (OR: 0.08; 95% CI: 0.02–0.33).

**Fig. 3:**
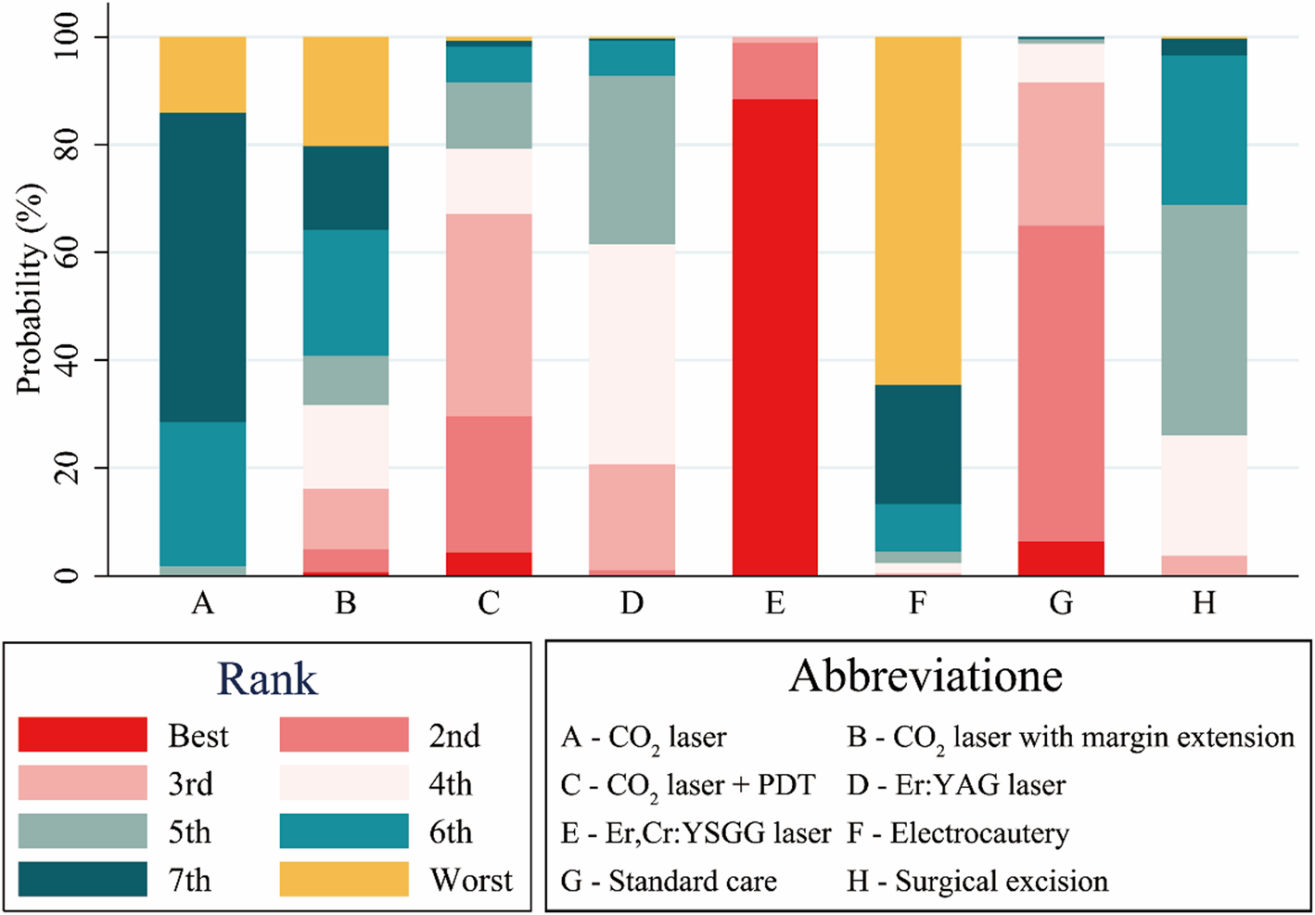
Ranking diagrams regarding network meta-analysis of the recurrence of OLK after different interventions

Figure 3 and Supplementary Table 7 illustrate the best probability ranking of interventions based on the recurrence outcome. Er,Cr:YSGG laser exhibited the highest probability of being ranked the best, followed by standard care and CO_2_ laser with photodynamic therapy (PDT). SUCRA values corroborated the best probability ranking, further supporting the superiority of Er,Cr:YSGG laser. The SUCRA values were consistent with the best probability ranking of the results, with Er,Cr:YSGG laser (97.8%), standard care (81.0%), CO_2_ laser combined with PDT (67.2%), Er:YAG laser (54.1%), surgical excision (42.0%), CO_2_ laser with margin extension (32.8%), CO_2_ laser (16.3%), and electrocautery (8.8%) (Supplementary Material Table 8 and Fig. S4). Thus, Er,Cr:YSGG laser emerged as the best intervention based on the number of postoperative recurrences in patients with OLK, while electrocautery ranked the least effective.

#### Comparison of the differences in intraoperative hemorrhage and postoperative pain scores on the first day after surgery across various management methods

**Fig. 4:**
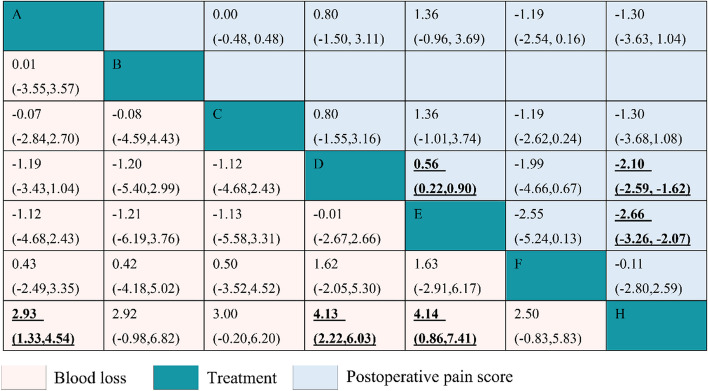
Results of the network meta-analysis of the differences in blood loss during surgery and postoperative pain score on the first day after surgery across various management methods

Figure 4 and Supplementary Figs. S5 and S6 present the results of pairwise comparisons for intraoperative hemorrhage and postoperative pain scores on the first day after surgery for different treatment modalities. Regarding intraoperative hemorrhage, 21 pairwise comparisons were made, three of which revealed statistically significant differences. Specifically, CO_2_ laser demonstrated significantly less bleeding than surgical excision (MD: 2.93; 95% CI: 1.33–4.54), as did Er:YAG laser (MD: 4.13; 95% CI: 2.22–6.03) and Er,Cr:YSGG laser (MD: 4.14; 95% CI: 0.86–7.41). The SUCRA values for bleeding ranked interventions in the following decreasing order: Er:YAG laser (76.7%), Er:Cr:YSGG laser (73.1%), CO_2_ laser combined with PDT (53.1%), CO_2_ laser with margin extension (51.3%), CO_2_ laser (49.9%), electrocautery (42.9%), and surgical excision (1.3%) (Supplementary Table S9 and Fig. S7). Therefore, Er:YAG laser and surgical excision ranked the best and worst regarding intraoperative hemorrhage for different treatment modalities in patients with OLK, respectively.

Regarding pain scores on the first day after surgery, 15 pairwise comparisons were performed, three of which demonstrated significant differences. Er,Cr:YSGG laser was found to be superior to both Er:YAG laser (MD: 0.56; 95% CI: 0.22–0.90) and surgical excision (MD: -2.66; 95% CI: − 3.26, − 2.07). Er:YAG laser was also associated with significantly lower pain scores compared to surgical excision (MD: -2.10; 95% CI: − 2.59, − 1.62). The SUCRA values for pain scores ranked interventions in the following descending order: Er,Cr:YSGG laser (93.8%), Er:YAG laser (67.9%), CO_2_ laser combined with PDT (54.2%), CO_2_ laser (54.1%), electrocautery (15.1%), and surgical excision (14.9%) (Supplementary Table 10 and Fig. S8). Consequently, Er,Cr:YSGG laser emerged as the most effective intervention in minimizing postoperative pain, while surgical excision was ranked the least effective.

### Publication bias

Funnel plots revealed no significant asymmetry regarding the number of postoperative recurrences, intraoperative hemorrhage, or postoperative pain scores across different treatment modalities (Supplementary Figs. S9-S11), suggesting a low likelihood of publication bias in the included studies.

### Quality assessment

**Fig. 5:**
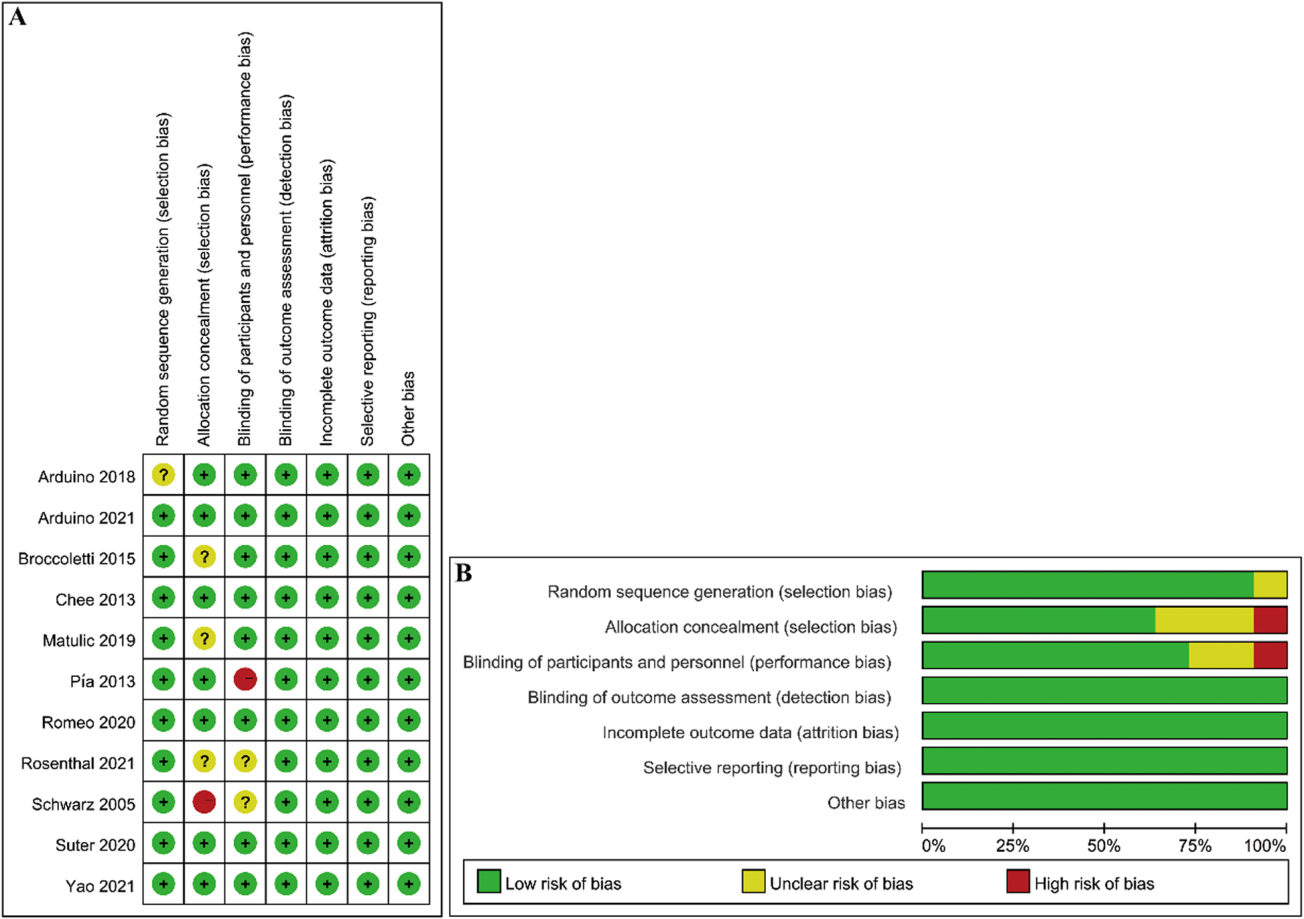
Results of risk bias

The quality of the included studies was assessed using the Cochrane Risk of Bias Tool. Figure 5 summarizes the results. Four of the 11 studies were judged to have a low risk of bias. Two studies were categorized as having a high risk of bias due to inadequate blinding of the participants and personnel and inadequate blinding of outcome assessment, respectively. The remaining five studies were associated with concerns regarding bias in certain domains.

## Discussion

### Summary of findings

According to the World Health Organization, OLK is one of the most common precancerous lesions since as early as 1972, being defined as “a morphologically altered tissue that has a greater potential for developing cancer than the corresponding normal-appearing tissue” [37]. While surgical excision remains the gold standard for managing OLK, the associated trauma and postoperative pain deter many patients, impacting their comfort and decreasing their willingness to seek treatment. Additionally, the recurrence rate after surgical intervention has been reported inconsistently [38]. Therefore, identifying appropriate treatment modalities for OLK and enhancing patient compliance are crucial for effectively preventing and intercepting the development of OSCC [39].

The present systematic review and network meta-analysis, encompassing 1138 OLK lesions in 917 patients, explored the similarities and differences in postoperative recurrence rates, intraoperative hemorrhage, and postoperative pain scores across various laser and conventional surgical interventions. The results revealed that Er,Cr:YSGG laser, CO_2_ laser combined with PDT, and Er:YAG laser demonstrated superior efficacy compared to surgical excision in preventing OLK recurrence. Additionally, all the laser-based treatments outperformed surgical excision in minimizing intraoperative hemorrhage and postoperative pain scores on the first postoperative day, with Er,Cr:YSGG and Er:YAG lasers demonstrating the highest rankings in these respective categories. Notably, none of the included studies reported severe adverse events with any treatment modality.

Currently, several lasers are employed to manage OLK, including CO_2_ lasers, erbium lasers (Er,Cr:YSGG with a wavelength of 2780 nm and Er:YAG with a wavelength of 2940 nm), and semiconductor lasers. CO_2_ lasers primarily function through vaporization and heat coagulation, leading to intraoperative pain and carbonized tissue remnants [40]. This thermal damage prevents the surgeon from accurately assessing the depth of involvement and compromises the postoperative aesthetic outcome [41]. Furthermore, CO_2_ laser therapy often elicits a more pronounced inflammatory response, characterized by increased neutrophil infiltration, compared to erbium-based treatments. The excessive neutrophil influx may contribute to neutrophil extracellular traps (NETs), promoting lesion recurrence [42, 43].

In contrast, erbium lasers rely on a combined thermal and mechanical mechanism [44]. The thermal effect is mediated by the laser energy’s absorption by water in the oral soft tissues, resulting in rapid vaporization and micro-explosions due to pressure exceeding tissue structural integrity, followed by a mechanical effect where surrounding tissues sequentially burst and disintegrate as the energy diffuses, achieving precise excision of the diseased tissue. The near-perfect overlap between the erbium laser’s wavelength and the water absorption peak in oral soft tissues minimizes collateral damage, allowing for more precise cutting compared to CO_2_ lasers [45].

Hypoxia-inducible factor-1α (HIF-1α), a glycoprotein with tyrosine kinase activity, plays a crucial role in OLK progression. Its expression is directly linked to cancer spread, larger post-surgical tumor size, and poorer differentiation in patients [46]. Consequently, HIF-1α levels are a vital predictor of postoperative recurrence after ablative surgery. Notably, erbium laser therapy has emerged as a promising strategy to combat this postoperative recurrence risk. Previous studies have shown that erbium laser irradiation significantly downregulates the expression of HIF-1α and pro-inflammatory cytokines and promotes keratinization [47, 48]. By stimulating fibroblast proliferation, erbium laser irradiation enhances wound healing, ultimately reducing postoperative recurrence rates [49]. Our findings coincide with this concept, as the erbium laser group exhibited fewer recurrences than the carbon dioxide laser group. This observation is also consistent with earlier meta-analyses [50, 51]. Furthermore, previous studies have demonstrated that erbium laser therapy induces coagulation at temperatures below protein denaturation, reducing intraoperative pain [52]. Our findings are consistent with previous research consistently ranking erbium lasers among the top two options for minimizing intraoperative hemorrhage and postoperative pain scores in patients with OLK.

PDT harnesses light-activated photosensitizers to generate reactive oxygen species (ROS), preferentially targeting diseased cells and disrupting their function. This targeted approach offers distinct advantages for managing OLK. Firstly, the specific wavelengths employed in PDT (630–800 nm) have limited tissue penetration, ideal for superficial lesions like those commonly found in OLK [53]. Secondly, PDT exhibits high tissue selectivity due to the enhanced uptake and prolonged retention of photosensitizer by malignant or potentially malignant tissues. This targeted delivery of ROS minimizes damage to surrounding healthy tissues, preserving facial aesthetics and oral function, which is particularly desirable for patients with OLK [54]. Furthermore, individuals aged 50–70 exhibit the highest incidence of OLK, who often have limitations associated with age and health, making them less inclined toward surgical procedures [55]. Fortunately, PDT presents a highly suitable alternative. Its advantages include ease of operation, flexibility in treatment numbers and lesion targeting, and generally good patient tolerance.

However, PDT alone exhibits lower efficacy in managing OLK. Previous meta-analyses have indicated that PDT alone is effective in OLK management but has no clear advantage over surgery [56, 57]. The diminished efficacy of PDT alone can be attributed to the poor penetration of the photosensitizer and the low absorption efficiency of the tissue. Combining laser therapy with PDT addresses these challenges. Laser irradiation significantly enhances the permeability of the diseased area, selectively amplifying the absorption of the photosensitizer by the affected oral mucosa. This combined approach also increases the laser penetration depth, improving treatment outcomes [58].

In addition, the recurrence of OLK following laser therapy or surgical excision alone is closely linked to the phenomenon of “regional cancerization,” which involves chronic exposure to carcinogens triggering widespread, underlying genetic alterations in the epithelium in a specific area, increasing the risk of multiple malignant or potentially malignant lesions, even if they appear normal histologically [59]. The ability of PDT to selectively eliminate lesion cells addresses this limitation of solitary laser treatment. In the present network meta-analysis, CO_2_ laser combined with PDT was associated with significantly lower postoperative recurrence rates compared to CO_2_ laser alone, without impacting intraoperative hemorrhage or postoperative pain. While published evidence on laser–PDT combinations remains limited, future studies with diverse laser types and elucidating their mechanism of action are crucial for expanding precise and effective treatment options for OLK.

The present systematic review and network meta-analysis focused primarily on homogeneous OLK, which comprised most of the 1138 cases included. Based on our findings, in these patients, Er,Cr:YSGG, Er:YAG laser, and CO_2_ laser combined with PDT showed potential advantages compared to conventional surgical excision and other laser types. The benefits might include reduced postoperative recurrence, less intraoperative hemorrhage, lower postoperative pain, and improved patient comfort. However, further research is necessary to confirm these preliminary findings. Specifically, a well-designed, large-scale, multi-arm randomized controlled trial is needed to directly compare the efficacy and cost-effectiveness of these interventions. Additionally, head-to-head trials are imperative to determine the relative effectiveness of different laser and PDT combinations.

### Strengths and limitations

The present network meta-analysis comprised the first comprehensive comparison of therapeutic efficacy for OLK with various lasers and surgical excisions. We employed a rigorous search strategy encompassing four major databases to capture the highest level of evidence, including recent RCTs. Unlike previous meta-analyses focusing solely on malignant transformation, which is influenced by complex factors beyond treatment, we prioritized two patient-related outcomes: post-treatment recurrence and treatment comfort. This choice reflects the understanding that addressing recurrence and minimizing discomfort directly impact patients’ treatment and clinical outcomes. We assessed the impact of different interventions on the quality of life by evaluating intraoperative hemorrhage and postoperative pain scores. Furthermore, the closed-loop structure of our reticulated meta-analysis enabled direct and indirect comparisons, offering a hierarchical ranking of treatment priorities.

However, limitations must be acknowledged. First, restricting the search to four databases increases the potential for publication bias. Second, variations in laser devices, brands, pulses, and powers within categories and inconsistent follow-up durations give rise to heterogeneity and potential bias. Additionally, we did not assess the economic implications of different interventions, which may influence real-world decisions. Therefore, a cautious interpretation of our findings is crucial.

## Conclusion

In conclusion, the present network meta-analysis suggests that for patients with homogeneous OLK, Er,Cr:YSGG, Er:YAG, and CO_2_ lasers combined with PDT show promise in reducing post-treatment recurrence and improving patient comfort compared to surgical excision and other laser types. These findings highlight the potential of these laser-based interventions as alternative treatment options. However, further high-quality, multi-arm RCTs are necessary to generate robust evidence and make informed clinical decisions. Ultimately, this study provides valuable insights into treatment modalities for OLK.

### Supplementary Information

**Supplementary Material 1.**

## Data Availability

All data generated or analysed during this study are included in this published article [and its supplementary information files].

## Abbreviations

OLK: Oral leukoplakia
OSCC: Oral squamous cell carcinoma
RCTs: Randomized controlled trials
PRISMA: Preferred Reporting Items for Systematic Reviews and Meta-Analyses
PICO: Participants, Intervention, Comparator, and Outcomes
RR: Ratio ratios
95% CI: 95% confidence intervals
SMD: Standardized mean difference
IF: Inconsistency factor
SUCRA: Areas under the cumulative ranking curve
NET: Neutrophil extracellular traps
HIF-1α: Hypoxia-inducible factor-1α
ROS: Reactive oxygen species

## References

[1] Clarkson E, Hadioonzadeh R, Peters SM (2024). Treatment of Oral dysplasia. Dent Clin N Am.

[2] Carrard VC, van der Waal I (2018). A clinical diagnosis of oral leukoplakia; a guide for dentists. Medicina Oral Patologia Oral Y Cirugia Bucal.

[3] Zhang C, Li B, Zeng X (2023). The global prevalence of oral leukoplakia: a systematic review and meta-analysis from 1996 to 2022. Bmc Oral Health.

[4] Villa A, Woo SB (2017). Leukoplakia-a diagnostic and management algorithm. J Oral Maxillofac Surg.

[5] Amagasa T, Yamashiro M, Uzawa N (2011). Oral premalignant lesions: from a clinical perspective. Int J Clin Oncol.

[6] Staines K, Rogers H (2017). Oral leukoplakia and proliferative verrucous leukoplakia: a review for dental practitioners. Br Dent J.

[7] Ishii J, Fujita K, Komori T (2003). Laser surgery as a treatment for oral leukoplakia. Oral Oncol.

[8] Dong Y, Chen Y, Tao Y (2019). Malignant transformation of oral leukoplakia treated with carbon dioxide laser: a meta-analysis. Lasers Med Sci.

[9] van der Hem PS, Nauta JM, van der Wal JE (2005). The results of CO_2_ laser surgery in patients with oral leukoplakia: a 25 year follow. Oral Oncol.

[10] Lesniewski A, Estrin N, Romanos GE (2022). Comparing the use of diode lasers to light-emitting diode phototherapy in Oral soft and hard tissue procedures: a literature review. Photobiomodulation Photomedicine and Laser Surgery.

[11] Tenore G, Mohsen A, Nuvoli A (2023). The impact of laser thermal effect on histological evaluation of Oral soft tissue biopsy: systematic review. Dentistry journal.

[12] Zwahlen M, Renehan A, Egger M (2008). Meta-analysis in medical research: potentials and limitations. Urologic Oncology-Seminars and Original Investigations.

[13] Guan J-Y, Luo Y-H, Lin Y-Y (2023). Malignant transformation rate of oral leukoplakia in the past 20 years: a systematic review and meta-analysis. J Oral Pathol Med.

[14] Acikel C (2009). Meta-analysis and its place in evidence based medicine. Klinik Psikofarmakoloji Bulteni-Bulletin of Clinical Psychopharmacology.

[15] Jansen, JP, Naci, H. Is network meta-analysis as valid as standard pairwise meta-analysis? It all depends on the distribution of effect modifiers. BMC Med 2013; 11(1):1-8. 10.1186/1741-7015-11-159 PMC3707819 23826681

[16] Ahn E, Kang H (2021). Concepts and emerging issues of network meta-analysis. Korean Journal of Anesthesiology.

[17] Nikolakopoulou A, Mavridis D, Furukawa TA (2018). Living network meta-analysis compared with pairwise meta-analysis in comparative effectiveness research: empirical study. Bmj-British Medical Journal.

[18] Page MJ, McKenzie JE, Bossuyt PM (2021). The PRISMA 2020 statement: an updated guideline for reporting systematic reviews. Bmj-British Medical Journal.

[19] Nasser M (2020). Cochrane handbook for systematic reviews of interventions. Am J Public Health.

[20] Martimbianco ALC, Sa KMM, Santos GM (2023). Most Cochrane systematic reviews and protocols did not adhere to the Cochranes risk of bias 2.0 tool. Rev Assoc Med Bras.

[21] Stogiannis D, Siannis F, Androulakis E. Heterogeneity in meta-analysis: a comprehensive overview. Int J Biostat. 2023; 10.1515/ijb-2022-0070 36961993

[22] Dettori JR, Norvell DC, Chapman JR (2022). Fixed-effect vs random-effects models for Meta-analysis: 3 points to consider. Global Spine Journal.

[23] Seitidis G, Nikolakopoulos S, Hennessy EA (2022). Network Meta-analysis techniques for synthesizing prevention science evidence. Prev Sci.

[24] Wang R, Dwan K, Showell MG (2022). Reporting of Cochrane systematic review protocols with network meta-analyses-a scoping review. Res Synth Methods.

[25] Kossmeier M, Tran US, Voracek M (2020). Charting the landscape of graphical displays for meta-analysis and systematic reviews: a comprehensive review, taxonomy, and feature analysis. BMC Med Res Methodol.

[26] Arduino PG, Cafaro A, Cabras M (2018). Treatment outcome of oral leukoplakia with Er: YAG laser: a 5-year follow-up prospective comparative study. Photomed Laser Surg.

[27] Arduino PG, Lodi G, Cabras M (2021). A randomized controlled trial on efficacy of surgical excision of nondysplastic leukoplakia to prevent oral cancer. Cancer Prev Res.

[28] Broccoletti R, Cafaro A, Gambino A (2015). Er:YAG laser versus cold knife excision in the treatment of nondysplastic Oral lesions: a randomized comparative study for the postoperative period. Photomed Laser Surg.

[29] Chee M, Sasaki C (2013). Carbon dioxide laser fiber for the excision of oral leukoplakia. Ann Otol Rhinol Laryngol.

[30] Lopez-Jornet P, Camacho-Alonso F (2013). Comparison of pain and swelling after removal of oral leukoplakia with CO_2_ laser and cold knife: a randomized clinical trial. Med Oral Patol Oral Cir Bucal.

[31] Matulić N, Bago I, Sušić M (2019). Comparison of Er:YAG and Er,Cr:YSGG Laser in the Treatment of Oral Leukoplakia Lesions Refractory to the Local Retinoid Therapy. Photobiomodulation, photomedicine, and laser surgery.

[32] Romeo U, Mohsen M, Palaia G (2020). CO_2_ laser ablation of oral leukoplakia: with or without extension of margins?. La Clinica terapeutica.

[33] Rosenthal M, Baser RE, Migliacci J (2021). Flexible fiber-based CO_2_ laser vs monopolar cautery for resection of oral cavity lesions: a single center randomized controlled trial assessing pain and quality of life following surgery. Laryngoscope investigative otolaryngology.

[34] Schwarz F, Maraki D, Yalcinkaya S (2005). Cytologic and DNA-cytometric follow-up of oral leukoplakia after CO_2_ and Er:YAG-laser assisted ablation: a pilot study. Lasers Surg Med.

[35] Suter VGA, Altermatt HJ, Bornstein MM (2020). A randomized controlled trial comparing surgical excisional biopsies using CO_2_ laser, Er:YAG laser and scalpel. Int J Oral Maxillofac Surg.

[36] Yao Y, Shi L, Wang Y (2021). Ablative fractional laser-assisted photodynamic therapy vs. ablative fractional laser for oral leukoplakia treatment: a randomized, controlled pilot study. Photodiagn Photodyn Ther.

[37] Farah CS (2021). Molecular, genomic and mutational landscape of oral leukoplakia. Oral Dis.

[38] Tangsuksan P, Chuerduangphui J, Yupanqui CT (2021). Mucoadhesive film containing α-mangostin shows potential role in oral cancer treatment. Bmc Oral Health.

[39] Kumari P, Debta P, Dixit A (2022). Oral potentially malignant disorders: etiology, pathogenesis, and transformation into Oral Cancer. Front Pharmacol.

[40] Condor D, Culcitchi C, Blum R (2021). A review of CO_2_ laser-mediated therapy for Oral mucosal lesions. Applied Sciences-Basel.

[41] Lopes-Santos G, Peralta-Mamani M, Oliveira DT (2023). Histological implications of high-power laser use in the oral soft tissue lesions: a systematic review. Lasers Med Sci.

[42] De Meo ML, Spicer JD (2021). The role of neutrophil extracellular traps in cancer progression and metastasis. Semin Immunol.

[43] Xiong S, Dong L, Cheng L (2021). Neutrophils in cancer carcinogenesis and metastasis. J Hematol Oncol.

[44] Apel C, Meister J, Ioana RS (2002). The ablation threshold of Er:YAG and Er:YSGG laser radiation in dental enamel. Lasers Med Sci.

[45] Malekafzali B, Asnaashari M, Javadi F (2017). Comparison of marginal microleakage of flowable composite restorations in primary canine teeth prepared with high-speed diamond bur, Er:YAG laser and Er,Cr:YSGG laser. Laser therapy.

[46] Rashid M, Zadeh LR, Baradaran B (2021). Up-down regulation of HIF-1α in cancer progression. Gene..

[47] Meyle J (2012). Mechanical, chemical and laser treatments of the implant surface in the presence of marginal bone loss around implants. European journal of oral implantology.

[48] Aoki A, Mizutani K, Schwarz F (2015). Periodontal and peri-implant wound healing following laser therapy. Periodontol.

[49] Feist IS, De Micheli G, Carneiro SR (2003). Adhesion and growth of cultured human gingival fibroblasts on periodontally involved root surfaces treated by Er:YAG laser. J Periodontol.

[50] Liu R, Sun K, Wang Y (2020). Clinical comparison between Er: YAG and CO_2_ laser in treatment of oral tumorous lesions: a meta-analysis. Medicine (Baltimore).

[51] Paglioni MP, Migliorati CA, Pereira Faustino IS (2020). Laser excision of oral leukoplakia: does it affect recurrence and malignant transformation? A systematic review and meta-analysis. Oral Oncol.

[52] Kong S, Aoki A, Iwasaki K (2018). Biological effects of Er:YAG laser irradiation on the proliferation of primary human gingival fibroblasts. J Biophotonics.

[53] Chamoli A, Gosavi AS, Shirwadkar UP (2021). Overview of oral cavity squamous cell carcinoma: risk factors, mechanisms, and diagnostics. Oral Oncol.

[54] Nasrin A, Hassan M, Gomes VG (2020). Two-photon active nucleus-targeting carbon dots: enhanced ROS generation and photodynamic therapy for oral cancer. Nanoscale..

[55] Foy J-P, Bertolus C, Saintigny P (2019). Oral cancer prevention worldwide: challenges and perspectives. Oral Oncol.

[56] Binnal A, Tadakamadla J, Rajesh G (2022). Photodynamic therapy for oral potentially malignant disorders: a systematic review and meta-analysis. Photodiagn Photodyn Ther.

[57] Zhang R, Gao T, Wang D (2023). Photodynamic therapy (PDT) for oral leukoplakia: a systematic review and meta-analysis of single-arm studies examining efficacy and subgroup analyses. Bmc Oral Health.

[58] Zhang Y, Zhang L, Yang D (2017). Treatment of oral refractory large area mucosal leukoplakia with CO_2_ laser combined with photodynamic therapy: case report. Photodiagn Photodyn Ther.

[59] Gabusi A, Gissi DB, Montebugnoli L (2020). Prognostic impact of intra-field heterogeneity in oral squamous cell carcinoma. Virchows Arch.

