# Supplementary material for: Laser therapy decreases oral leukoplakia recurrence and boosts patient comfort: a network meta-analysis and systematic review
Source: BMC Oral Health. 2024 Apr 17;24:469. doi: 10.1186/s12903-024-04179-9 (PMC11025167; doi:10.1186/s12903-024-04179-9)
Supplement: Supplementary file 1 — Supplementary Material 1. [file 12903_2024_4179_MOESM1_ESM.docx]

**Supplementary information**

**Table S1** Detailed search strategy for each database 2

**Table S2** The general characteristics of all included studies 5

**Figure S1** the results of pairwise meta-analysis 8

**Table S3** Results of a local inconsistency test for the number of OLK recurrences after different intervention treatments 9

**Table S4** Results of a local inconsistency test for the bleeding volume after different intervention treatments 9

**Table S5** Results of a local inconsistency test for the pain scores after different intervention treatments 10

**Figure S2** Loop inconsistency detection results 11

**Figure S3** Forest plot of the network meta-analysis of the OLK recurrences after different interventions 12

**Table S6** Results of the network meta-analysis of the number of OLK recurrences after different interventions 12

**Table S7** Probability ranking results table for recurrence 13

**Table S8** SUCRA rank for recurrence 13

**Figure S4** SUCRA rank Graph for recurrence 13

**Figure S5** Forest plot of the network meta-analysis of bleeding volume after different interventions 14

**Figure S6** Forest plot of the network meta-analysis of pain score after different interventions 14

**Table S9** SUCRA rank table for bleeding volume 15

**Figure S7** SUCRA rank Graph for bleeding volume 15

**Table S10** SUCRA rank table for pain score 16

**Figure S8** SUCRA rank Graph for pain score 16

**Figure S9** Funnel plots for recurrence 17

**Figure S10** Funnel plots for bleeding volume 17

**Figure S11** Funnel plots for pain score 18

**Table S1** Detailed search strategy for each database

| **PubMed** | |  |
| --- | --- | --- |
| #3 | Search: ("leukoplakia"[MeSH Terms] OR Leukoplakia[Text Word] (Leukoplakias, Oral) OR Oral Leukoplakia OR Oral Leukoplakias OR (Leukokeratosis, Oral) OR (Leukokeratoses, Oral) OR Oral Leukokeratoses OR Oral Leukokeratosis OR (Keratosis, Oral) OR (Keratoses, Oral ) OR Oral Keratoses OR Oral Keratosis) AND ("randomized controlled trial"[Publication Type] OR "randomized controlled trials as topic"[MeSH Terms] OR "randomized controlled trials"[All Fields] OR (Clinical Trials, Randomized) OR (Trials, Randomized Clinical) OR (Controlled Clinical Trials, Randomized)) Sort by: Most Recent |  |
| #2 | Search: "randomized controlled trial"[Publication Type] OR "randomized controlled trials as topic"[MeSH Terms] OR "randomized controlled trials"[All Fields] OR (Clinical Trials, Randomized) OR (Trials, Randomized Clinical) OR (Controlled Clinical Trials, Randomized) Sort by: Most Recent |  |
| #1 | Search: "leukoplakia"[MeSH Terms] OR Leukoplakia[Text Word] (Leukoplakias, Oral) OR Oral Leukoplakia OR Oral Leukoplakias OR (Leukokeratosis, Oral) OR (Leukokeratoses, Oral) OR Oral Leukokeratoses OR Oral Leukokeratosis OR (Keratosis, Oral) OR (Keratoses, Oral ) OR Oral Keratoses OR Oral Keratosis Sort by: Most Recent |  |
| **EMBASE** | |  |
| #11 | (#1 OR #2 OR #3 OR #4 OR #5 OR #6 OR #7 OR #8 OR #9 OR #10) AND [randomized controlled trial]/lim |  |
| #10 | 'oral keratosis' |  |
| #9 | 'oral keratoses' |  |
| #8 | 'keratoses, oral' |  |
| #7 | 'oral leukokeratoses' |  |
| #6 | 'leukokeratoses, oral' |  |
| #5 | '(leukokeratosis, oral)' |  |
| #4 | 'oral leukoplakias' |  |
| #3 | 'oral leukoplakia' |  |
| #2 | 'leukoplakias, oral' |  |
| #1 | 'leukoplakias, oral' |  |
| **Web of Science** | | |
| #8 | #7 AND #4 and Preprint Citation Index | |
| #7 | (((((TS=(randomized controlled trial)) OR TS=(randomized controlled trials as topic)) OR TS=(randomized controlled trials)) OR TS=(Clinical Trials, Randomized)) OR TS=(Trials, Randomized Clinical)) OR TS=(ontrolled Clinical Trials, Randomized) and Preprint Citation Index | |
| #6 | #5 AND #4 and Preprint Citation Index | |
| #5 | ((((((TS=(photochemotherapy)) OR TS=(Photodynamic therapy)) OR TS=(Photochemotherapies)) OR TS=(Photodynamic Therapy)) OR TS=(Therapy, Photodynamic)) OR TS=(Photodynamic Therapies)) OR TS=(Therapies, Photodynamic) and Preprint Citation Index | |
| #4 | #3 OR #2 OR #1 and Preprint Citation Index | |
| #3 | (((((TS=(Oral Leukoplakia)) OR TS=(Oral Leukoplakias)) OR TS=(Leukokeratosis, Oral)) OR TS=(Leukokeratoses, Oral)) OR TS=(Oral Leukokeratoses)) OR TS=(Oral Leukokeratosis) and Preprint Citation Index | |
| #2 | TS=(Leukoplakias, Oral) and Preprint Citation Index | |
| #1 | TS=((Leukoplakias, Oral)) and Preprint Citation Index | |
| **Cochrane Library database** | | |
| #24 | #16 AND #23 | |
| #23 | #17 OR #18 OR #19 OR #20 OR #21 OR #22 | |
| #22 | (Controlled Clinical Trials, Randomized) | |
| #21 | (Trials, Randomized Clinical) | |
| #20 | (Clinical Trials, Randomized) | |
| #19 | randomized controlled trials | |
| #18 | randomized controlled trials as topic | |
| #17 | randomized controlled trial | |
| #16 | #1 OR #2 OR #3 OR #4 OR #5 OR #6 OR #7 OR #8 OR #9 OR #10 OR #11 OR #12 OR #13 OR #14 OR #15 | |
| #15 | Oral Keratosis | |
| #14 | Oral Keratoses | |
| #13 | (Keratoses, Oral ) | |
| #12 | (Keratosis, Oral) | |
| #11 | "Oral Leukokeratosis" | |
| #10 | Oral Leukokeratosis | |
| #9 | Oral Leukokeratoses | |
| #8 | (Leukokeratoses, Oral) | |
| #7 | (Leukokeratosis, Oral) | |
| #6 | "Oral Leukoplakias" | |
| #5 | Oral Leukoplakias | |
| #4 | (Leukoplakias, Oral) | |
| #3 | "Oral Leukoplakia" | |
| #2 | Oral Leukoplakia | |
| #1 | ((Leukoplakias, Oral)):ti,ab,kw (Word variations have been searched) | |

**Table S2** The general characteristics of all included studies

| Study | Gender | No. of patients | No. of lesions | Site | Interventions | Study period | Endpoints |
| --- | --- | --- | --- | --- | --- | --- | --- |
| Schwarz 2005 | Male (5)  Female (5) | 10 | 16 homogeneous | Gingiva (9)  Tongue (2)  Floor of the mouth (3)  Angle of mouth (2) | Er:YAG laser (8)  CO_2_ laser (8) | 24 months | Therapeutical remission, recurrence, malignant conversion after treatment |
| Chee 2013 | NA | 45 | 47 (NA) | NA | Surgical excision (23)  CO_2_ laser (24) | 22 months | Recurrence, surgical excision time, blood loss |
| Pía 2013 | Male (27)  Female (21) | 48 | 46 homogeneous,  2 heterogeneous | Gingiva (24)  Tongue (3)  Buccal mucosa (10)  Palate (8)  Floor of the mouth (3) | Surgical excision (28)  CO_2_ laser (20) | 40 months | Recurrence, malignant conversion after treatment, blood loss, postoperative pain and swelling assessment |
| Broccoletti 2015 | Male (146)  Female (198) | 344 | 394 (NA) | Gingiva (100)  Tongue (110)  Buccal mucosa (116)  Palate (44)  Floor of the mouth (24) | Surgical excision (221)  Er:YAG laser (173) | 26 months | Recurrence, operative time, Postoperative pain, swelling, and life quality assessment. |
| Arduino 2018 | Male (40)  Female (47) | 87 | 117 homogeneous | Gingiva (48)  Tongue (18)  Buccal mucosa (45)  Palate (6) | Surgical excision (58)  Er:YAG laser (59) | 84 months | Recurrence |
| Matulic 2019 | Male (16)  Female (38) | 54 | 54 homogeneous | Gingiva (22)  Buccal mucosa (14)  Floor of the mouth (18) | Er:YAG laser (27)  Er,Cr:YSGG laser (27) | 17 months,  follow-up  12months | Recurrence, blood loss, postoperative pain assessment |
| Romeo 2020 | Male (14)  Female (16) | 19 | 22(NA) | Gingiva (7)  Tongue (6)  Buccal mucosa (3)  Palate (3)  Floor of the mouth (3) | CO_2_ laser (11)  CO_2_ laser with margin extension (11) | Follow-up  6 months | The elimination of associated risk factors, recurrence, blood loss |
| Suter 2020 | Male (41)  Female (33) | 74 | 74 homogeneous | Buccal mucosa (74) | CO_2_ laser (24)  Er:YAG laser (25)  Surgical excision (25) | Follow-up  6 months | Recurrence, operative time, blood loss, postoperative pain and swelling assessment, histopathological slide examination |
| Arduino 2021 | Male (128)  Female (132) | 130 | 260 homogeneous | Tongue (63)  Buccal mucosa (76)  Palate (121) | Surgical excision (130)  Standard care (130) | Follow-up  12 months | Recurrence, malignant conversion after treatment, adverse reaction |
| Rosenthal 2021 | Male (29)  Female (33) | 62 | 62 homogeneous | Tongue (48)  Buccal mucosa (1)  Floor of the mouth (13) | Electrocautery (30)  CO_2_ laser (32) | 96 months | Recurrence, blood loss, operative time, postoperative pain, swelling, and life quality assessment |
| Yao 2021 | Male (23)  Female (21) | 44 | 32 homogeneous  12 heterogeneous | NA | CO_2_ laser (21)  CO_2_ laser - PDT (23) | Follow-up  12months | Recurrence, blood loss, operative time, postoperative pain, swelling, and life quality assessment |


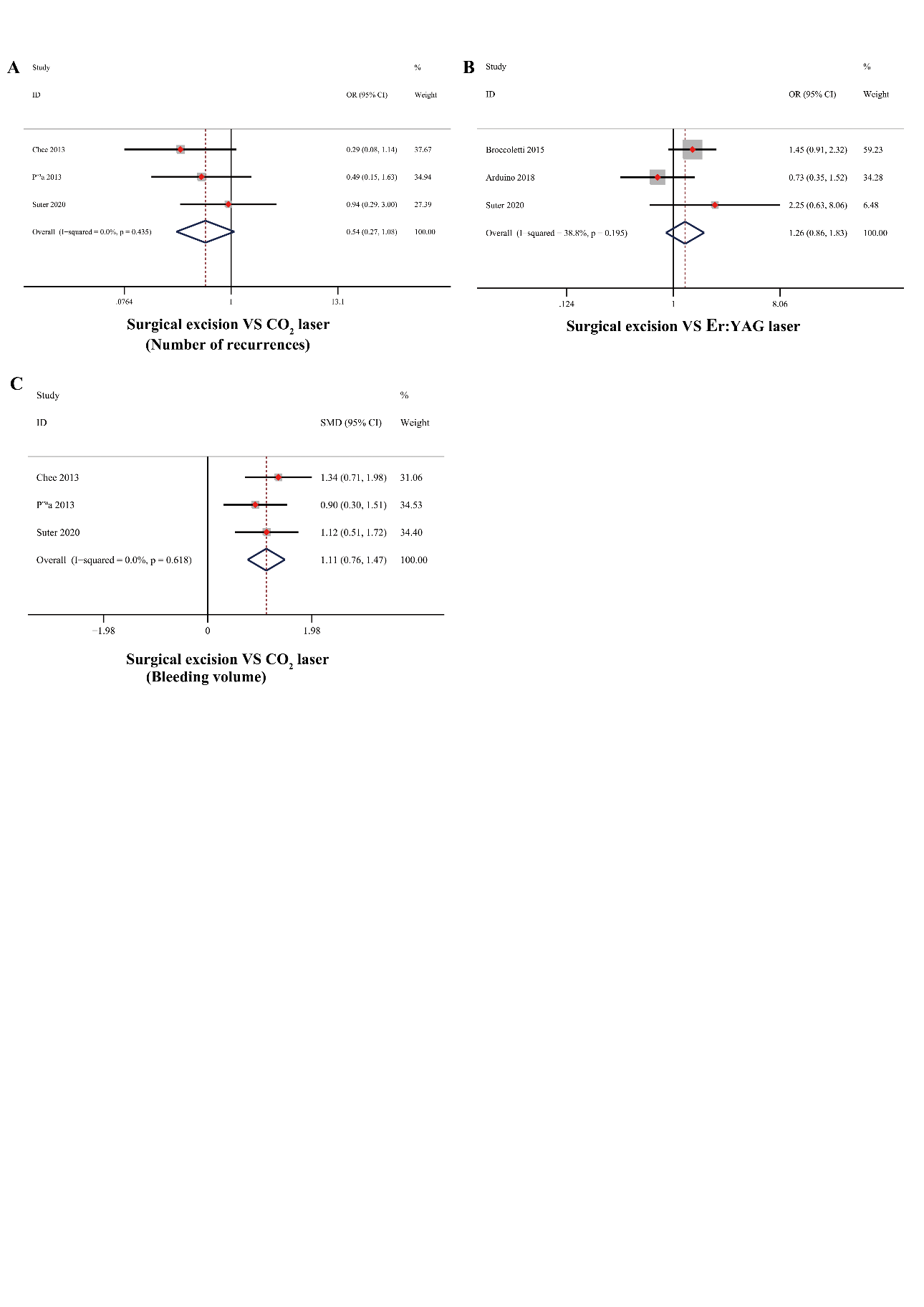


**Figure S1** the results of pairwise meta-analysis

**Table S3** Results of a local inconsistency test for the number of OLK recurrences after different intervention treatments

| Side | Direct |  | Indirect |  | Difference |  |  | tau |
| --- | --- | --- | --- | --- | --- | --- | --- | --- |
|  | Coef. | Std. Err. | Coef. | Std. Err. | Coef. | Std. Err. | P>z |  |
| A B | . | . | . | . | . | . | . | . |
| A C | . | . | . | . | . | . | . | . |
| A D | -.9754768 | .6597864 | -.8944402 | .5688123 | -.0810366 | .8783078 | 0.926 | .2908949 |
| A F | . | . | . | . | . | . | . | . |
| A H | -.6205245 | .3949136 | -1.585243 | 1.110412 | .9647185 | 1.175465 | 0.412 | .2678785 |
| D E | -2.302585 | .6716985 | -.4535526 | 200.1178 | -1.849033 | 200.1169 | 0.993 | .2098477 |
| D H | .2140691 | .2506927 | -.2201655 | 1.243197 | .4342346 | 1.265282 | 0.731 | .238411 |
| G H | 1.255266 | .5686023 | -2.704155 | 199.8561 | 3.959421 | 199.8583 | 0.984 | .2098491 |

**Table S4** Results of a local inconsistency test for the bleeding volume after different intervention treatments

| Side | Direct |  | Indirect |  | Difference |  |  | tau |
| --- | --- | --- | --- | --- | --- | --- | --- | --- |
|  | Coef. | Std. Err. | Coef. | Std. Err. | Coef. | Std. Err. | P>z |  |
| A H | 1.511576 | .8631978 | 11.06733 | 11.06733 | -9.555755 | 3.780391 | 0.061 | 1.365317 |
| A B | .01 | 1.814787 | 5.863213 | 186.925 | -5.853213 | 186.9291 | 0.975 | 1.2578 |
| A C | -.07 | 1.413398 | 5.860503 | 131.419 | -5.930503 | 131.4285 | 0.964 | 2.672497 |
| A D | .0004071 | 2.687733 | -2.301806 | 3.267973 | 2.302213 | 4.231306 | 0.586 | .286211 |
| A F | .43 | 1.489598 | 5.877004 | 199.0458 | -5.447004 | 199.0517 | 0.978 | 1.25779 |
| D H | 4.326479 | 1.059141 | 1.464726 | 3.44448 | 2.861754 | 3.580646 | 0.424 | 1.335574 |
| D E | -.01 | 1.359766 | 8.253729 | 112.6189 | -8.263729 | 112.6287 | 0.942 | 1.257776 |

**Table S5** Results of a local inconsistency test for the pain scores after different intervention treatments

| Side | Direct |  | Indirect |  | Difference |  |  | tau |
| --- | --- | --- | --- | --- | --- | --- | --- | --- |
|  | Coef. | Std. Err. | Coef. | Std. Err. | Coef. | Std. Err. | P>z |  |
| A C | . | . | . | . | . | . | . | . |
| A D | -1.087914 | 1.266054 | .533484 | 2.525954 | -1.621398 | 2.708978 | 0.549 | 1.43e-08 |
| A F | . | . | . | . | . | . | . | . |
| A H | . 2.36322 | 1.937256 | -.3093524 | 2.594991 | 2.672572 | 3.834148 | 0.486 | 7.55e-07 |
| D E | -.56 | .1734722 | 1.617673 | 39.60388 | -2.177673 | 39.60397 | 0.956 | 4.39e-07 |
| D H | 2.099041 | .2485068 | 3.490207 | 6.414018 | -1.391166 | 6.427002 | 0.829 | 3.36e-06 |


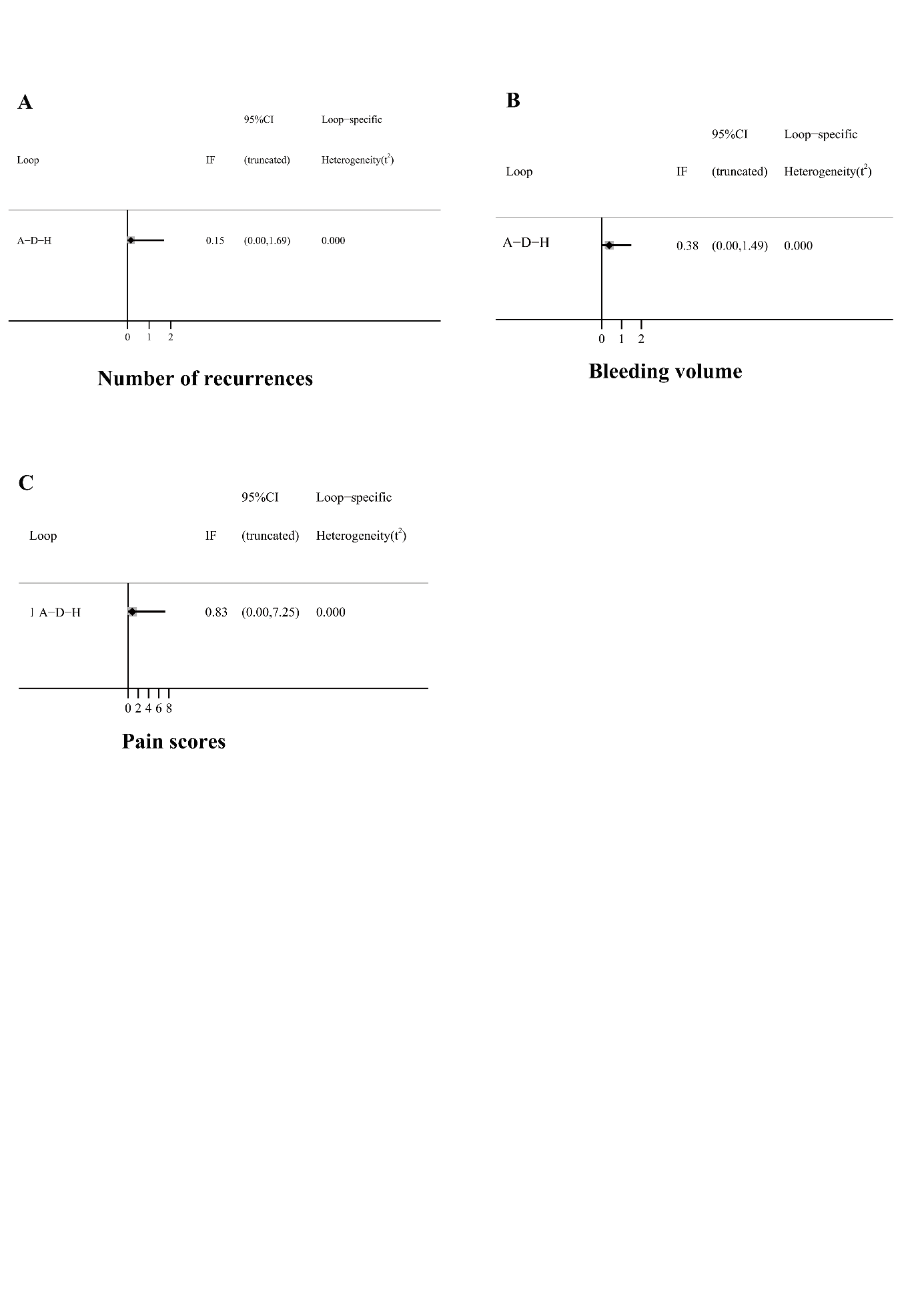


**Figure S2** Loop inconsistency detection results


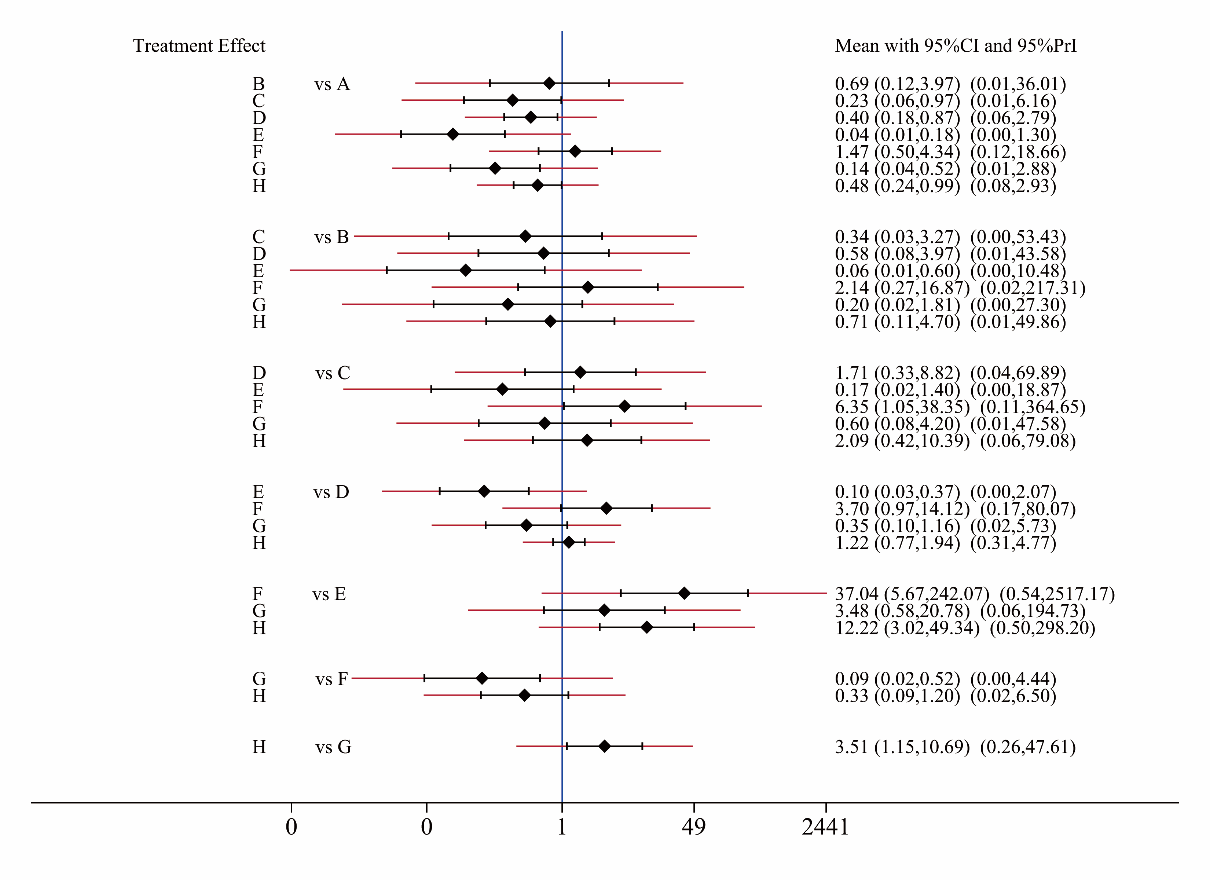


**Figure S3** Forest plot of the network meta-analysis of the OLK recurrences after different interventions. Black shapes and solid lines represent the OR and corresponding 95% CI for each comparison, respectively. blue line indicates no effect

**Table S6** Results of the network meta-analysis of the number of OLK recurrences after different interventions

| E |  |  |  |  |  |  |  |
| --- | --- | --- | --- | --- | --- | --- | --- |
| 0.29 (0.05,1.71) | G |  |  |  |  |  |  |
| 0.17 (0.02,1.40) | 0.60 (0.08,4.20) | C |  |  |  |  |  |
| **0.10 (0.03,0.37)** | 0.35 (0.10,1.16) | 0.58 (0.11,3.00) | D |  |  |  |  |
| **0.08 (0.02,0.33)** | 0.29 (0.09,0.87) | 0.48 (0.10,2.37) | 0.82 (0.51,1.30) | H |  |  |  |
| **0.06 (0.01,0.60)** | 0.20 (0.02,1.81) | 0.34 (0.03,3.27) | 0.58 (0.08,3.97) | 0.71 (0.11,4.70) | B |  |  |
| **0.04 (0.01,0.18)** | **0.14 (0.04,0.52)** | **0.23 (0.06,0.97)** | **0.40 (0.18,0.87)** | **0.48 (0.24,0.99)** | 0.69 (0.12,3.97) | A |  |
| **0.03 (0.00,0.18)** | **0.09 (0.02,0.52)** | **0.16 (0.03,0.95)** | 0.27 (0.07,1.03) | 0.33 (0.09,1.20) | 0.47 (0.06,3.67) | 0.68 (0.23,2.01) | F |

**Table S7** Probability ranking results table for recurrence

| Study and rank | Treatment | | | | | | | | | | |  |
| --- | --- | --- | --- | --- | --- | --- | --- | --- | --- | --- | --- | --- |
|  | A | B | C | D | E | F | | G | | H | |  |
| Best | 0.0 | 0.3 | 3.7 | 0.0 | 88.0 | | 0.0 | | 8.0 | | 0.0 | |
| 2nd | 0.0 | 4.0 | 26.1 | 1.0 | 9.2 | | 0.0 | | 59.7 | | 0.0 | |
| 3rd | 0.0 | 9.8 | 39.4 | 19.6 | 2.5 | | 0.5 | | 25.8 | | 2.4 | |
| 4th | 0.2 | 14.6 | 10.3 | 44.5 | 0.3 | | 1.9 | | 4.9 | | 23.3 | |
| 5th | 2.0 | 9.6 | 12.0 | 27.6 | 0.0 | | 2.6 | | 1.3 | | 44.9 | |
| 6th | 24.3 | 25.4 | 6.3 | 6.5 | 0.0 | | 11.8 | | 0.2 | | 25.5 | |
| 7th | 58.6 | 16.2 | 1.1 | 0.6 | 0.0 | | 20.3 | | 0.1 | | 3.1 | |
| Worst | 14.9 | 20.1 | 1.1 | 0.2 | 0.0 | | 62.9 | | 0.0 | | 0.8 | |

**Table S8** SUCRA rank for recurrence

| Treatment | SUCRA | PrBest | MeanRank |
| --- | --- | --- | --- |
| A B C D E F G H | 16.3 32.8 67.2 54.1 97.8 8.8 81.0 42.0 | 0.0 0.3 3.7 0.0 88.0 0.0 8.0 0.0 | 6.9 5.7 3.3 4.2 1.2 7 7.4 2.3 5.1 |


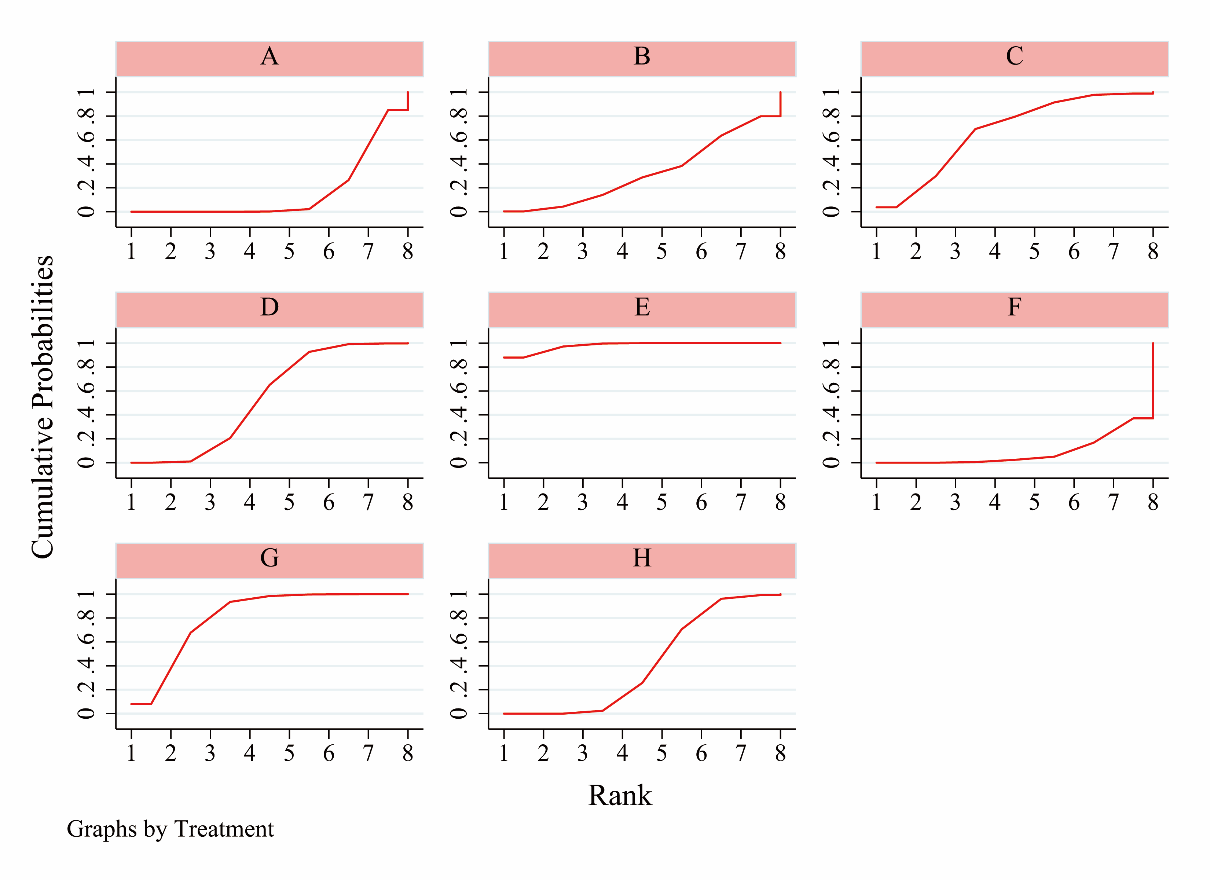


**Figure S4** SUCRA rank Graph for recurrence


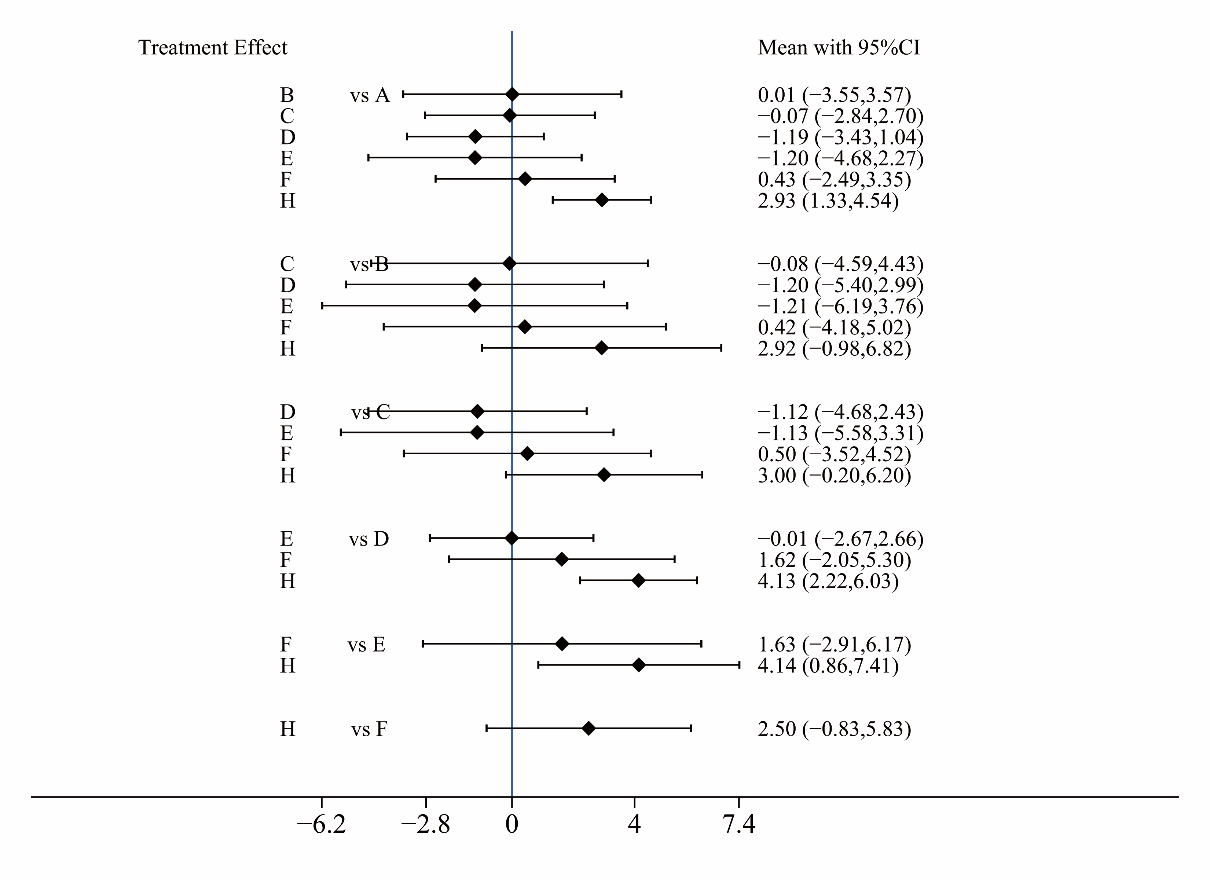


**Figure S5** Forest plot of the network meta-analysis of bleeding volume after different interventions. Black shapes and solid lines represent the SMD and corresponding 95% CI for each comparison, respectively. blue line indicates no effect


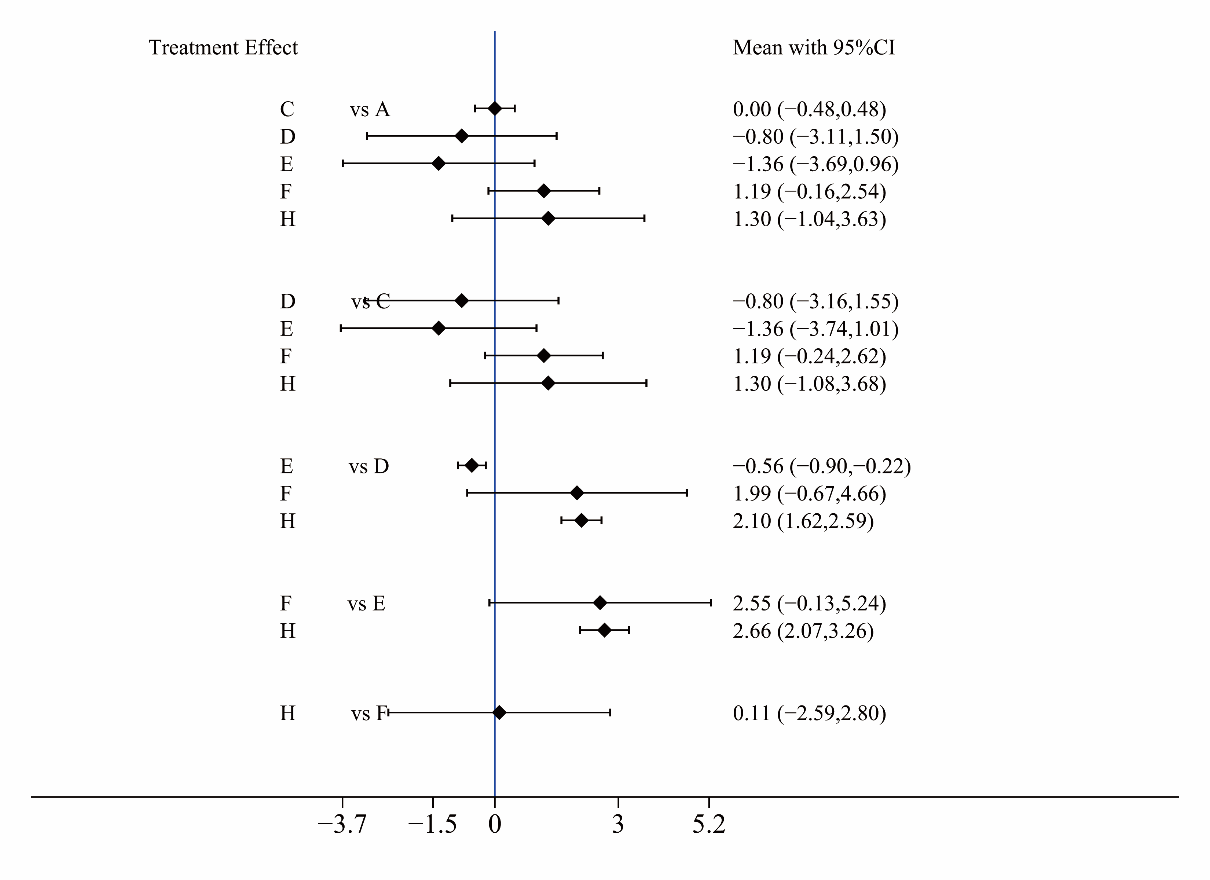


**Figure S6** Forest plot of the network meta-analysis of pain score after different interventions. Black shapes and solid lines represent the SMD and corresponding 95% CI for each comparison, respectively. blue line indicates no effect

**Table S9** SUCRA rank table for bleeding volume

| Treatment | SUCRA | PrBest | MeanRank |
| --- | --- | --- | --- |
| A B C D E F H | 49.9 51.3 53.1 76.7 73.1 42.9 3.1 | 1.4 16.9 13.4 24.2 36.1 8.1 0.0 | 4.0 3.9 3.8 2.4 2.6  4.4  6.8 |


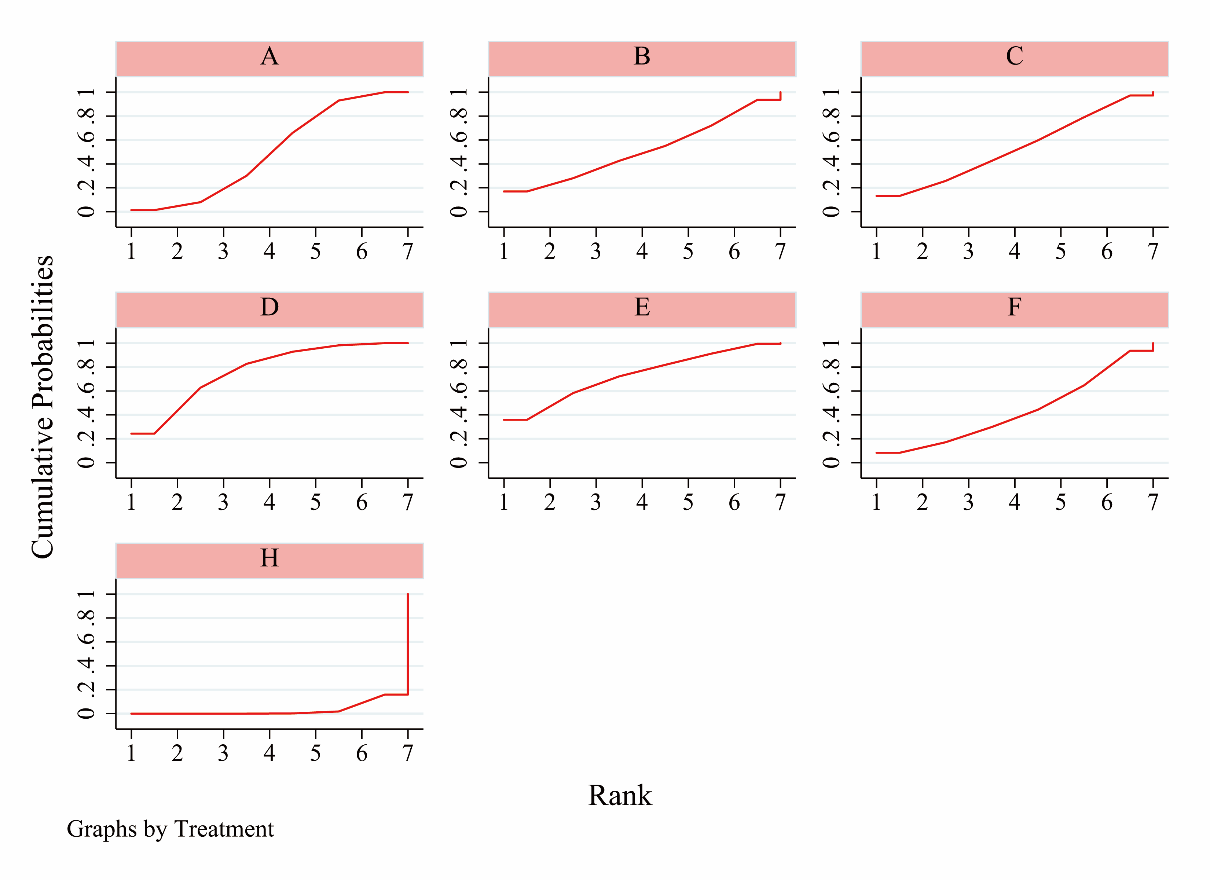


**Figure S7** SUCRA rank Graph for bleeding volume

**Table S10** SUCRA rank table for pain score

| Treatment | SUCRA | PrBest | MeanRank |
| --- | --- | --- | --- |
| A C D E F H | 54.1 54.2 67.9 93.8 15.1 14.9 | 6.7 8.7 0.0 84.2 0.4 0.0 | 3.3 3.3 2.6 1.3 5.2  5.3 |


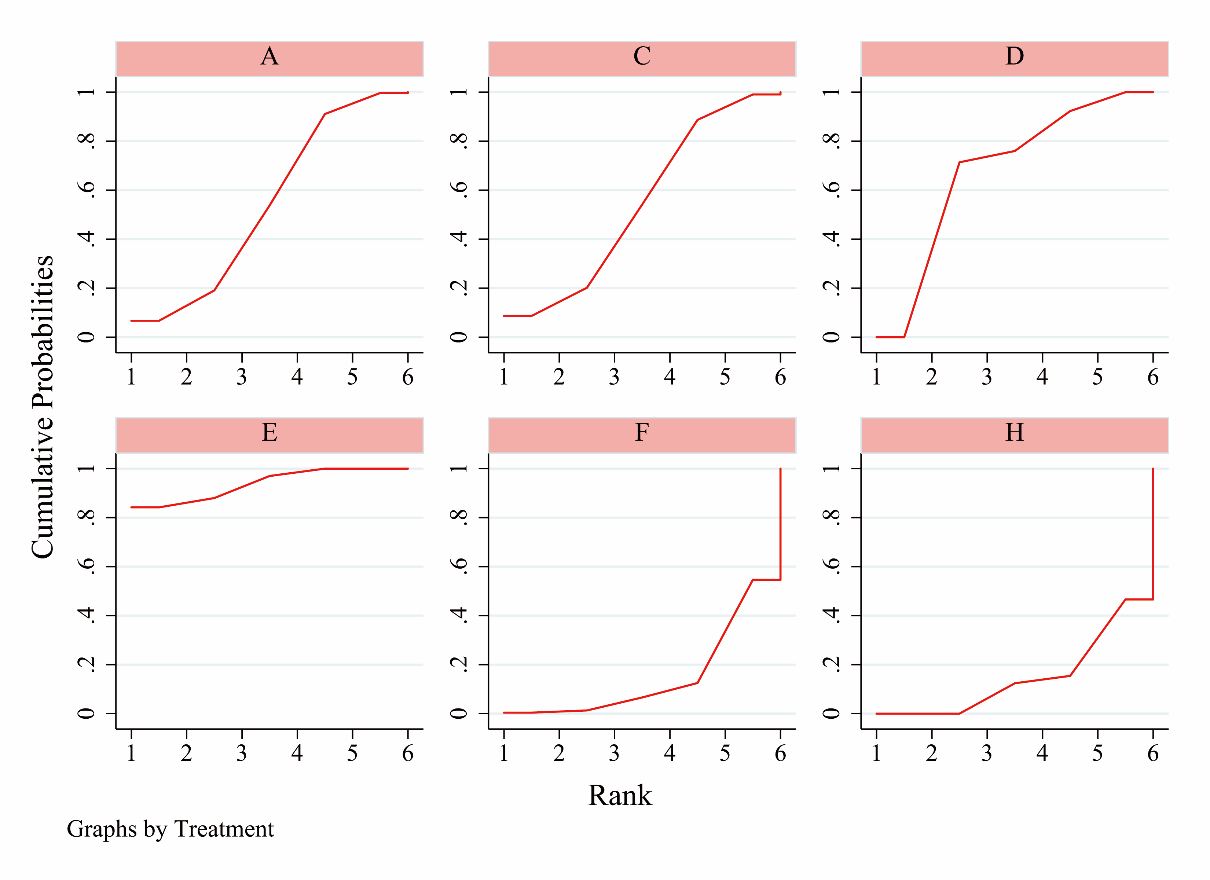


**Figure S8** SUCRA rank Graph for pain score


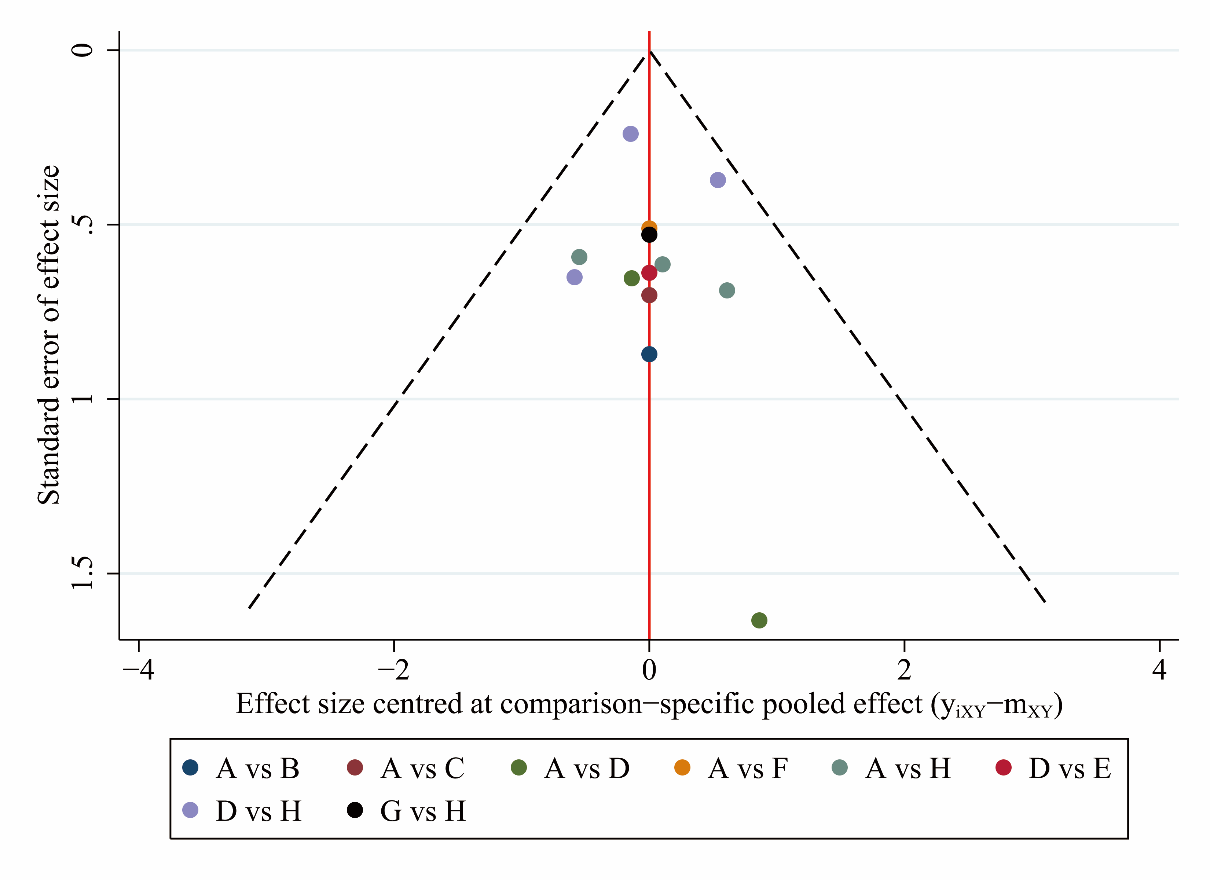


**Figure S9** Funnel plots for recurrence


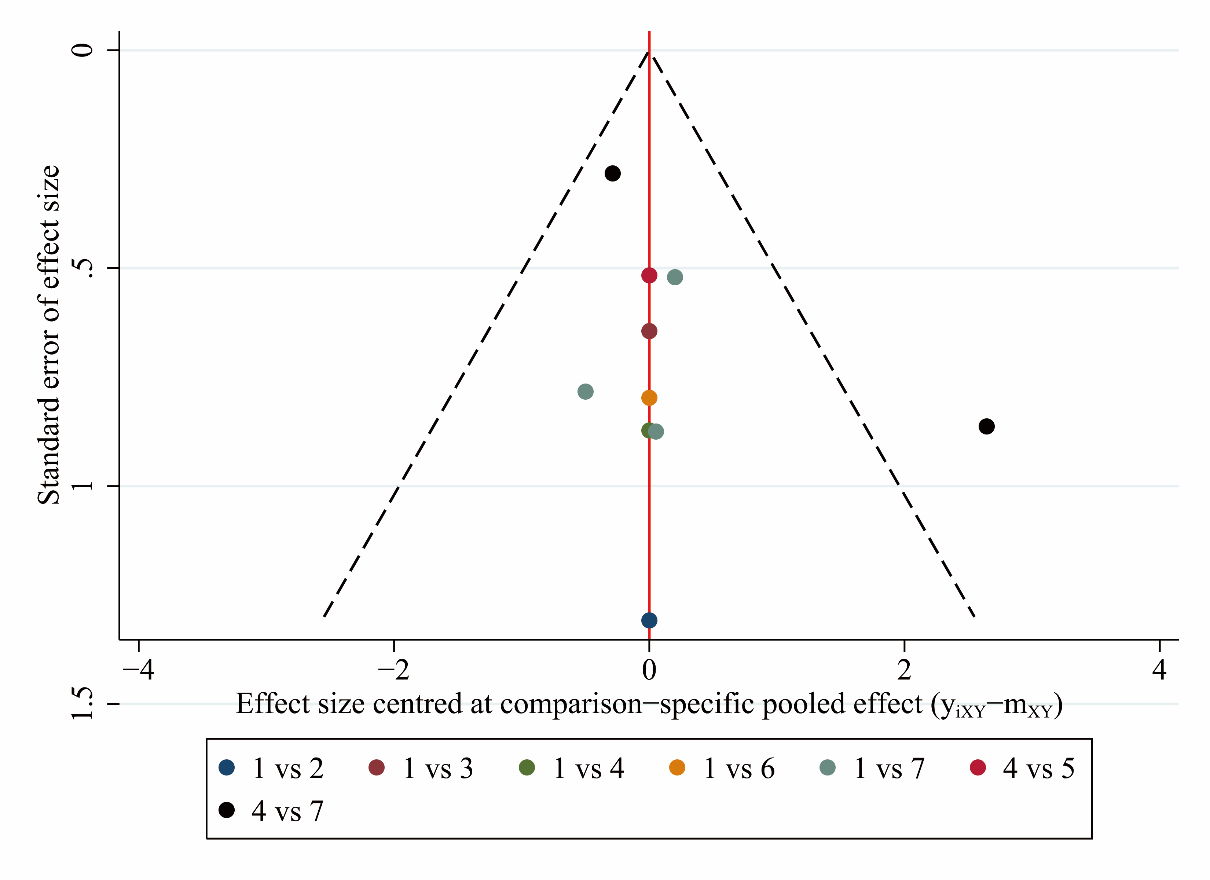


**Figure S10** Funnel plots for bleeding volume


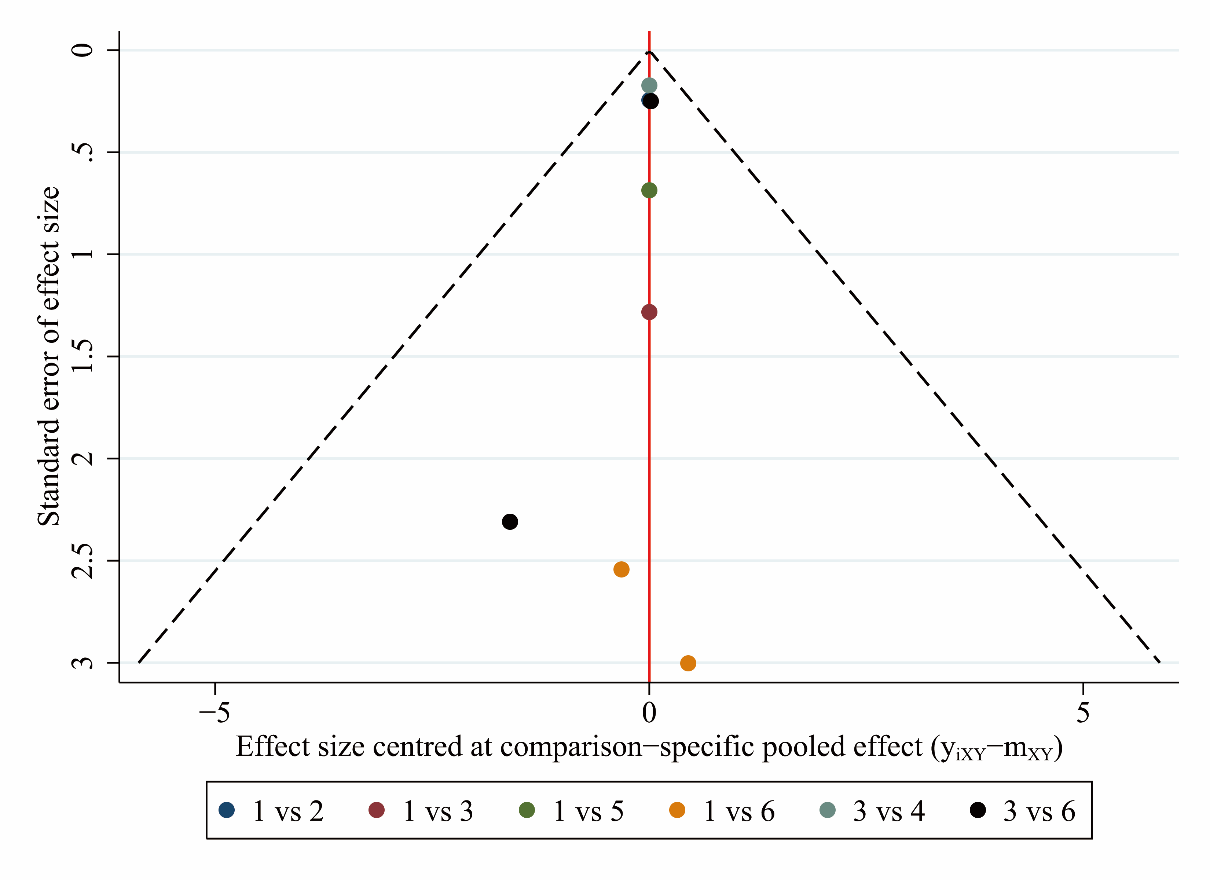


**Figure S11** bleeding volume for pain score
